# Prognostic and clinical pathological significance of the systemic immune-inflammation index in urothelial carcinoma: a systematic review and meta-analysis

**DOI:** 10.3389/fonc.2024.1322897

**Published:** 2024-03-26

**Authors:** Yao Wang, Xiaoming Hao, Gang Li

**Affiliations:** ^1^ Department of Urology, Heji Hospital Affiliated to Changzhi Medical College, Changzhi, China; ^2^ Department of Urology, Heping Hospital Affiliated to Changzhi Medical College, Changzhi, China

**Keywords:** urothelial carcinoma, systemic immune-inflammation index, SII, prognostic value, survival outcomes, meta-analysis

## Abstract

**Background:**

A new non-invasive biomarker, the Systemic Immune-Inflammation Index (SII), has been proven to have prognostic value in multiple cancers. This systematic review and meta-analysis aimed to investigate the prognostic and clinical pathological significance of SII in urothelial carcinoma.

**Methods:**

A comprehensive search was conducted across multiple databases, including PubMed, Web of Science, Embase, Cochrane Library, and CNKI. The quality of the included studies was assessed using the Newcastle-Ottawa Scale (NOS). Hazard ratios (HR) with 95% confidence intervals (CI) were calculated to evaluate the prognostic value of SII before treatment on survival outcomes, and odds ratios (OR) with 95%CI were used to assess the correlation between SII before treatment and clinical pathological features.

**Results:**

This meta-analysis included a total of 10 studies (11 datasets) with 6,333 patients. The pooled analysis showed that high SII before surgery was significantly associated with poor survival outcomes in patients with urothelial carcinoma, including overall survival (OS) (HR=1.55, 95%CI 1.24-1.95, p<0.001), cancer-specific survival (CSS) (HR=2.74, 95%CI 1.67-4.49, p<0.001), recurrence-free survival (RFS) (HR=2.74, 95%CI 1.67-4.49, p<0.001), and progression-free survival (PFS) (HR=1.66, 95%CI 1.36-2.02, p<0.001). In addition, patients with elevated preoperative SII values were more likely to have adverse pathological features, including larger tumor size and advanced pathological T stage (p<0.001).

**Conclusion:**

These findings suggest a significant association between high SII levels before treatment and poor survival outcomes, as well as certain clinical pathological features, in patients with urothelial carcinoma.

## 1 Introduction

Urothelial carcinoma (UC) encompasses renal pelvis carcinoma, ureter carcinoma, bladder carcinoma (BC), and urethral carcinoma, and represents a multifocal malignant tumor affecting the urinary system (1–3). UC has a relatively high incidence rate, with BC being the most common (4). In European and American populations, upper tract urothelial carcinoma (UTUC) accounts for approximately 5% to 10% of all urinary tract urothelial carcinomas (5). In China, the incidence of UTUC is notably higher compared to Western populations (6). Despite both UTUC and BC originating from the urothelium, they arise from distinct embryonic tissues, leading to variations in their occurrence and progression processes (7, 8). UTUC exhibits higher invasiveness, with around two-thirds of UTUC patients presenting invasive characteristics at the time of diagnosis (7). The SEER database reports a global 5-year cancer-specific survival rate of 50% for UTUC patients. In contrast, approximately 50% of urethral cancer cases are secondary to UTUC or BC. Therefore, early assessment of the prognostic risk in UC patients and intervention targeting the corresponding risk factors are of paramount importance in improving the survival outcomes of urothelial carcinoma patients, particularly those with UTUC.

In recent years, non-invasive immune-inflammatory markers have emerged as potential parameters for predicting tumor prognosis. Among these markers, easily interpretable and effective inflammatory biomarkers have gained attention in predicting treatment outcomes and patient prognosis (9). These biomarkers include hematological markers such as the neutrophil-to-lymphocyte ratio (NLR) (10), lymphocyte-to-monocyte ratio (LMR) (11), platelet-to-lymphocyte ratio (PLR) (12), and systemic immune-inflammation index (SII) (13).

SII is calculated based on the peripheral blood platelet (P), neutrophil (N), and lymphocyte (L) counts, with the formula: P×N/L (14). Initial reports have shown that SII can predict the prognosis of patients with hepatocellular carcinoma after radical resection, and it has been identified as a powerful prognostic indicator for poor prognosis in hepatocellular carcinoma patients (15, 16). Subsequent studies have confirmed its effectiveness in predicting the prognosis of various cancer patients, including hepatocellular carcinoma (16), pancreatic cancer (17), gallbladder cancer (18), cholangiocarcinoma (19), gastric cancer (20), laryngeal cancer (21), endometrial cancer (22), non-small cell lung cancer (23), and bladder cancer (24). In recent years, the prognostic value of SII in urological system cancers has been investigated and confirmed by many researchers, although the conclusions are not entirely consistent (25–28). To the best of our knowledge, there has been no systematic review and meta-analysis specifically focused on UC, and the prognostic value of SII in UC and its relationship with clinical pathology remains unclear. Therefore, we conducted a comprehensive meta-analysis based on domestic and foreign databases to determine the prognostic value and clinical pathological significance of SII in UC patients.

## 2 Materials and methods

### 2.1 Ethics statement

This meta-analysis was conducted by the Preferred Reporting Items for Systematic Reviews and Meta-Analyses (PRISMA) statement (29). All data used in this meta-analysis were obtained from previously published studies; therefore, ethical approval and patient consent were not required for this study.

### 2.2 Search strategy

To minimize selection bias, we conducted comprehensive and detailed searches in four English databases (PubMed, Web of Science, Embase, and Cochrane Library) and one Chinese database (China National Knowledge Infrastructure, CNKI). The following search terms and text were utilized: (“systemic immune-inflammation index” OR “SII”) and (“urothelial carcinoma” OR “bladder carcinoma” OR “bladder cancer” OR “upper tract urothelial cancer” OR “upper tract urothelial carcinoma” OR “urethral carcinoma” OR “urethral cancer”) (**Supplementary Table S1**). The final search was updated in September 2023. Additionally, the reference lists of the included studies were manually searched to identify any potential studies that met the inclusion criteria. There were no language restrictions applied during the search process.

### 2.3 Inclusion and exclusion criteria

Inclusion and Exclusion Criteria The included studies had to meet the following inclusion criteria (1): patients with a postoperative pathological diagnosis of UC; (2) articles reporting the prognostic outcomes of pre-treatment SII, including overall survival (OS), cancer-specific survival (CSS), recurrence-free survival (RFS), or progression-free survival (PFS); (3) articles providing hazard ratios (HR) and 95% confidence intervals (CI) for survival outcomes; (4) a cut-off or threshold value for SII was determined, and the samples were divided into high and low groups based on SII values; (5) SII was calculated using the formula: P × N/L.

Studies were excluded if they met the following criteria: (1) duplicate articles; (2) reviews, comments, case reports, letters, conference abstracts, notes, or meta-analyses; (3) animal experiments; (4) insufficient or missing data for analysis; (5) low-quality assessment score (NOS < 7);

### 2.4 Data extraction and quality assessment

Two investigators independently conducted a thorough review and assessment of all the included studies, which involved literature screening, data extraction, and quality assessment. In the event of any disagreements or discrepancies, a third investigator was consulted, and a consensus was reached through discussion. Data were extracted from the eligible studies, including the first author’s name, publication year, region, study period, sample size, gender, age, cancer type, survival outcome measures, treatment methods, SII cut-off value or threshold, follow-up time, and survival outcome hazard ratios (HR) with corresponding 95% confidence intervals (CI). If both univariate and multivariate analysis data were provided in the articles, HR and 95% CI data were extracted from the multivariate analysis. The quality of all included articles was assessed using the Newcastle-Ottawa Scale (NOS) to evaluate the selection, comparability, and outcome of the studies (30). The scoring system ranged from 0 to 9, with a score of ≤6 indicating a high risk of bias and a score of >6 indicating a low risk of bias, indicating high-quality articles (**Supplementary Table S2**). Any disagreements that arose during this process were resolved through consensus among all investigators.

### 2.5 Statistical analysis

We performed a meta-analysis to calculate pooled hazard ratios (HR) and 95% confidence intervals (CI) to investigate the prognostic value of SII on survival outcomes in UC patients. The heterogeneity among the included studies was assessed using the Cochrane Q test and I^2^ statistics. A significance level of P<0.10 or I^2^>50% was considered indicative of significant heterogeneity, and a random-effects model was used in such cases. Subgroup analyses were conducted to explore potential sources of heterogeneity, including region, sample size, cancer type, treatment modality, SII cut-off value, and NOS score. These subgroup analyses aimed to identify any confounding factors that may influence the results. For binary variables, odds ratios (OR) and their corresponding 95% CI were calculated to determine the correlation between SII and clinical pathology. If the pooled OR > 1 (95% CI does not overlap 1), it indicates a correlation between high SII and poorer clinical pathological outcomes, otherwise, there is no correlation. Publication bias was assessed using a funnel plot, and the use of Begg’s test and Egger’s test. In the presence of bias, the Duval and Tweedie non-parametric trim and fill methods were employed to explore the potential influence of missing studies. Sensitivity analysis was performed by sequentially excluding individual datasets to evaluate the reliability and stability of the meta-analysis results. All statistical analyses were conducted using Stata 17.0 software (Stata Corp LP, College Station, TX, USA). Two-sided tests were used for all statistical analyses, and a p-value < 0.05 was considered statistically significant.

## 3 Results

### 3.1 Search results

**Figure 1:**
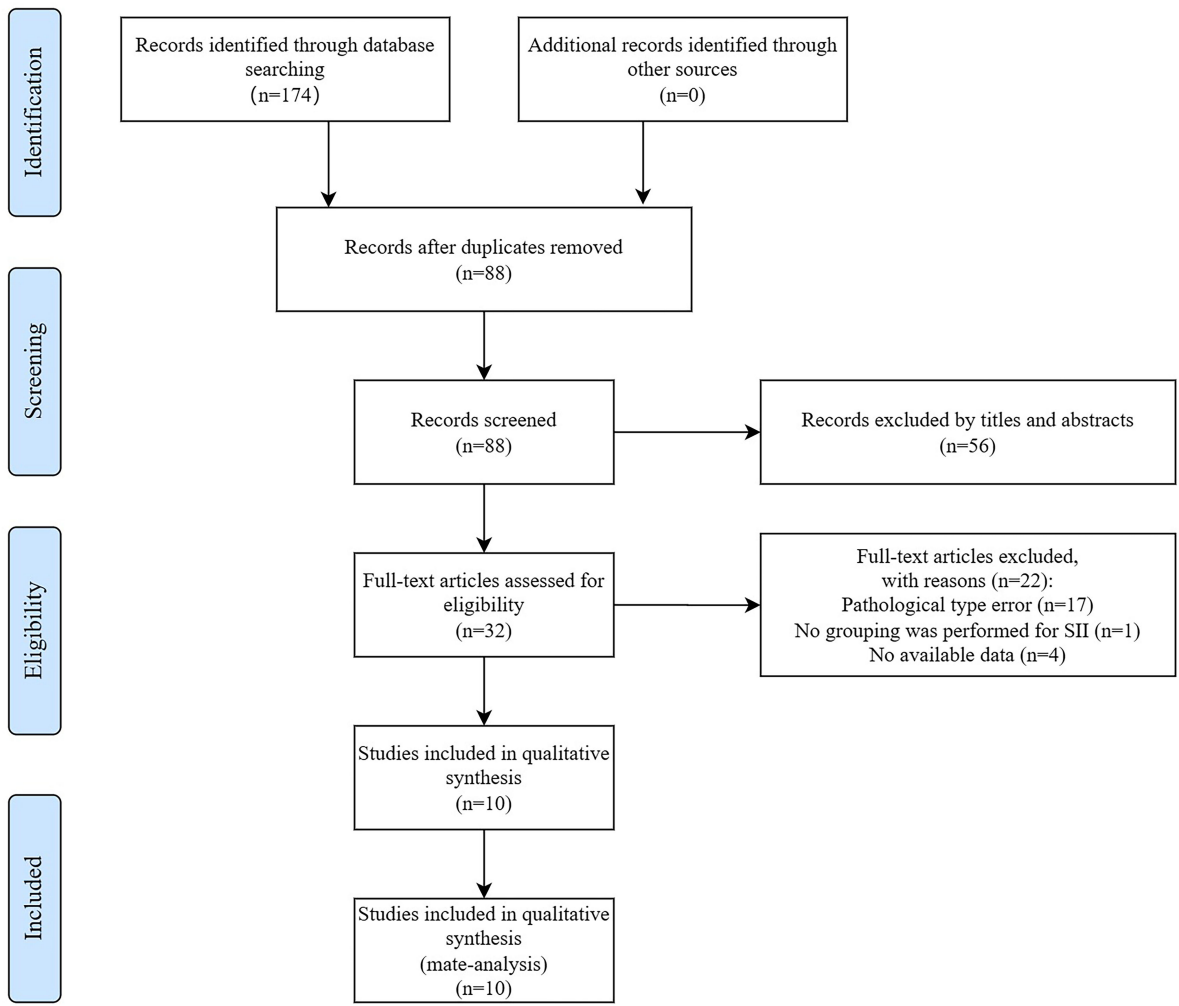
Flow diagram of included studies for this meta-analysis.

We initially retrieved a total of 174 studies from 5 databases. After removing 86 duplicate studies and conducting screening based on titles and abstracts, an additional 56 records were excluded. Subsequently, 32 potentially eligible articles were selected for full-text evaluation. Among these, 22 articles were excluded due to reasons such as insufficient data, lack of relevance to the topic, endpoints, and grouping. Finally, a total of 10 studies (11 datasets) met the inclusion criteria for this study, including 1 study from a Chinese database. All included studies were retrospective. The process of literature selection is visually presented in **Figure 1**

### 3.2 Characteristics of the included studies

The characteristics of the included studies are summarized in **Table 1**. These studies were retrospective and were published between 2019 and 2022. Among the 10 studies (11 datasets) included in our analysis, nine were published in English (31–39), and one study was published in Chinese (40). The studies were conducted in various countries, including China (31–33, 37, 39, 40), Austria (34), Europe and the United States (35), Slovakia (36), and Japan (38). The sample sizes ranged from 103 to 2372 participants. Regarding the survival outcomes, eight studies reported on the relationship between SII and overall survival (OS) (31–34, 36–39), seven studies reported on the relationship between SII and cancer-specific survival (CSS) (31–35, 37, 38), six studies examined the relationship between SII and recurrence-free survival (RFS) (32–35, 39, 40), and four studies investigated the relationship between SII and progression-free survival (PFS) (31, 35–37). The included studies focused on UTUC and BC, and the treatment modalities varied, including surgical treatment and chemotherapy. The cut-off values or ranges of SII ranged from 410.3 to 1326, with only one study not providing the number of patients in the SII groups [34]. The follow-up time was represented by the median or mean follow-up time. All the studies included in this analysis reported HR and 95% CI for survival outcomes, which were derived from multivariable analysis. No adjustment or receiver operating characteristic curve analysis was necessary in this study.

**Table 1:** Basic characteristics of the included studies.

Author	Year	Region	Study period	Sample	Sex	Age	Cancer	Survive	Treatment	Cut-off	No.of patients	Follow-up	NOS
				size	size		type	type		value	with high/low SII	(month)	
Jan (31)	2019	China	2007-2017	424	189/235	Median:70	UTUC	OS,CSS,PFS	Surgery	580	215/209	Median:35	8
Zheng (1) (32)	2020	China	2006-2015	253(TC)	180/73	Mean:67.6	UTUC	OS,CSS,RFS	Surgery	672.44	107/146	Median:33.8	9
Zheng(2) (32)	2020	China	2004-2016	272(VC)	182/90	Mean:65.9	UTUC	OS,CSS,RFS	Surgery	672.44	107/165	Median:44.6	9
Chien (33)	2021	China	2001-2013	376	146/230	Median:69.0	UTUC	MFS,CSS,BRFS	Surgery	485	210/166	Median:52.0	8
Mori (34)	2021	Austria	1990-2008	2373	1597/776	Median:69	UTUC	OS,CSS,RFS	Surgery	485	986/1387	Median:38.0	9
Katayama (35)	2021	US and Europe	1996-2007	1117	855/262	Median:67	BC	OS, CSS, PFS, RFS	Surgery	580	309/808	Median:64	9
Palacka (36)	2021	Slovakia	2000-2015	181	135/46	Median:66	MUC	OS,PFS	Chemotherapy	1326	NE	Median:9.5	7
Jan# (37)	2022	China	2008-2019	399	169/230	Mean:69.2	UTUC	OS,CSS,PFS	Surgery	580	184/215	Mean:49.2	8
Kobayashi (38)	2022	Japan	2004-2020	103	67/36	Median:73	UTUC	OS,CSS	Surgery	520	52/51	Median:41	9
Zhang (39)	2022	China	2010-2020	725	621/104	Median:65	BC	OS, RFS	Surgery	554.23	258/467	Median:36	8
Zhang* (40)	2022	China	2014-2020	110	57/53	Mean:66	UTUC	RFS	Surgery	410.3	53/57	Median:24	8

TC, training cohort; VC, validation cohort; UTUC, upper tract urothelial carcinoma; BC, bladder carcinoma; MUC, metastatic urothelial carcinoma; OS, overall survival; CSS, cancer-specific survival; PFS, progression-free survival; RFS, recurrence-free survival; NE, not expound; (1)(2): the same article two data sets; #: the same author as Jan; *: not the same author as zhang

### 3.3 Meta-analysis and subgroup analysis of different survival outcomes

#### 3.3.1 Impact of the SII for OS in urothelial carcinoma

**Figure 2:**
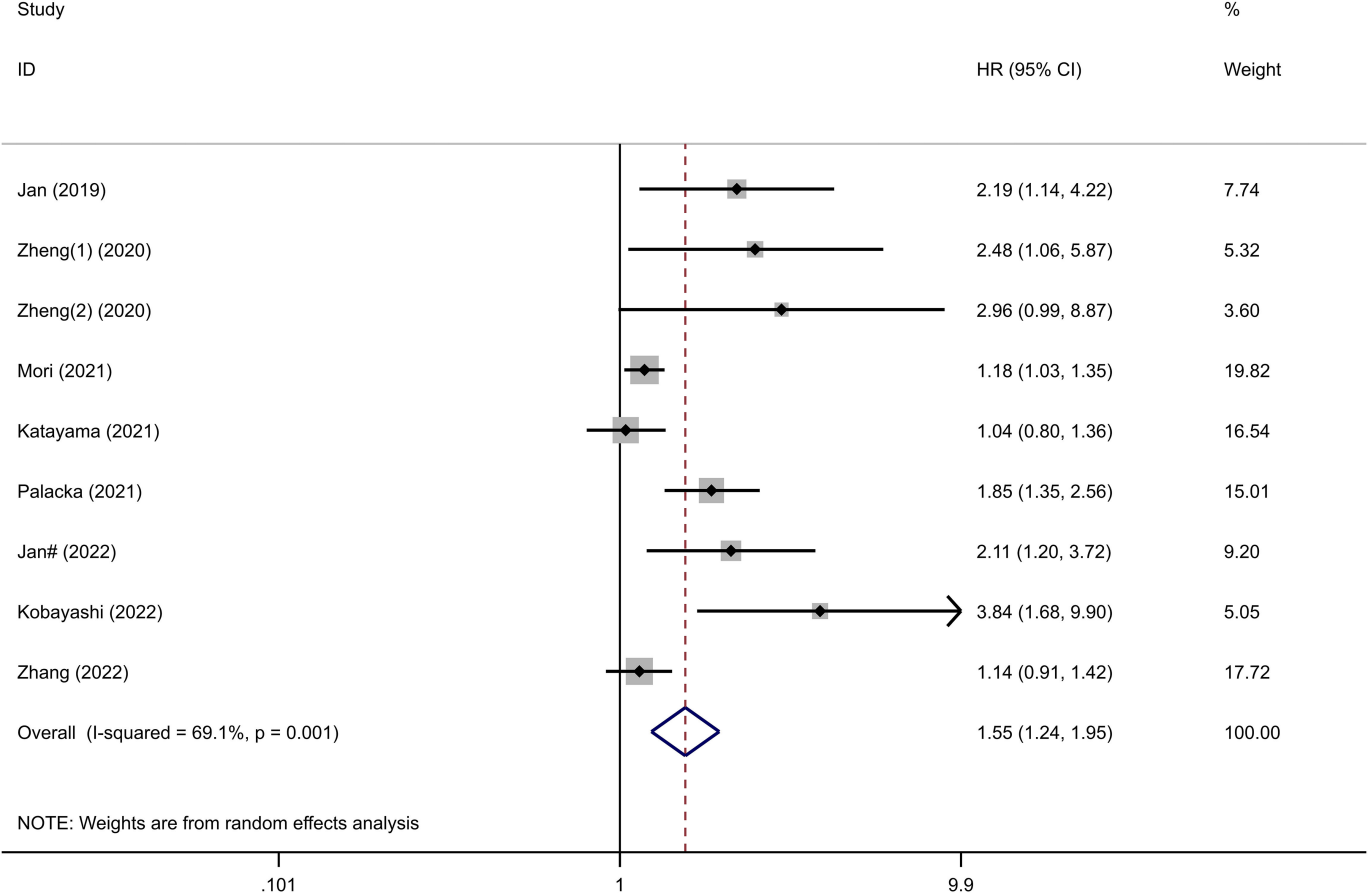
Forest plots showing the association between SII and OS in UC.

Eight studies comprising 5847 patients mentioned SII’s performance in predicting the OS of patients with UC. The pooled analysis revealed a significant association between elevated preoperative SII and poor OS (HR=1.55, 95%CI 1.24-1.95, p<0.001), as depicted in **Figure 2**. However, substantial heterogeneity was observed among the studies (I^2 ^= 69.1%, p=0.001), and a random-effects model was applied. To explore the potential sources of heterogeneity, subgroup analyses were conducted (**Supplementary Figure S1**). As shown in the subgroup analysis, SII had no significant prognostic value in studies where the cancer type was bladder cancer (HR= 1.10, 95% CI 0.93 -1.30, p=0.284), increased SII significantly predicted poor OS, regardless of region, sample size, cancer type, treatment modality, SII cut-off value, and NOS score. Further details of the subgroup analysis for OS are presented in **Table 2**.

**Table 2:** The subgroup analysis of the prognostic role of SII for OS in UC.

Variables	No.of studies	Sample	HR (95% CI)	P	Heterogeneity		Model
					I^2^ (%)	P	
**OS**							Random
Total	9	5847	1.55 (1.24-1.95)	<0.001	69.1	0.001	
**Region**							Random
China	5	2073	1.849 (1.200-2.849)	0.005	62.2	0.032	
No-China	4	3774	1.44 (1.04-2.00)	0.028	79.2	0.002	
**Sample**							Random
<300	4	809	2.11 (1.60-2.77)	<0.001	0	0.406	
≥300	5	5038	1.24 (1.03-1.50)	0.02	52.5	0.077	
**Cancer**							Random
UTUC	6	3824	2.09 (1.33-3.28)	0.001	71.1	0.004	
BC	2	1842	1.10 (0.93-1.30)	0.284	0	0.603	
MUC	1	181	1.85 (1.34-2.55)	<0.001	–	–	
**Treatment**							Random
Surgery	8	5666	1.49 (1.17-1.89)	0.001	65.4	0.005	
No-Surger	1	181	1.85 (1.34-2.55)	<0.001	–	–	
**Cut-off**							Random
≤580	6	3176.12	1.37 (1.09-1.73)	0.008	66.8	0.01	
>580	3	2670.88	1.98 (1.48-2.64)	<0.001	0	0.619	
**NOS**							Random
<9	4	1729	1.66 (1.16-2.38)	0.006	69.8	0.019	
≥9	5	4118	1.53 (1.07-2.18)	0.019	69.6	0.011	

#### 3.3.2 Impact of the SII for CSS in urothelial carcinoma

**Figure 3:**
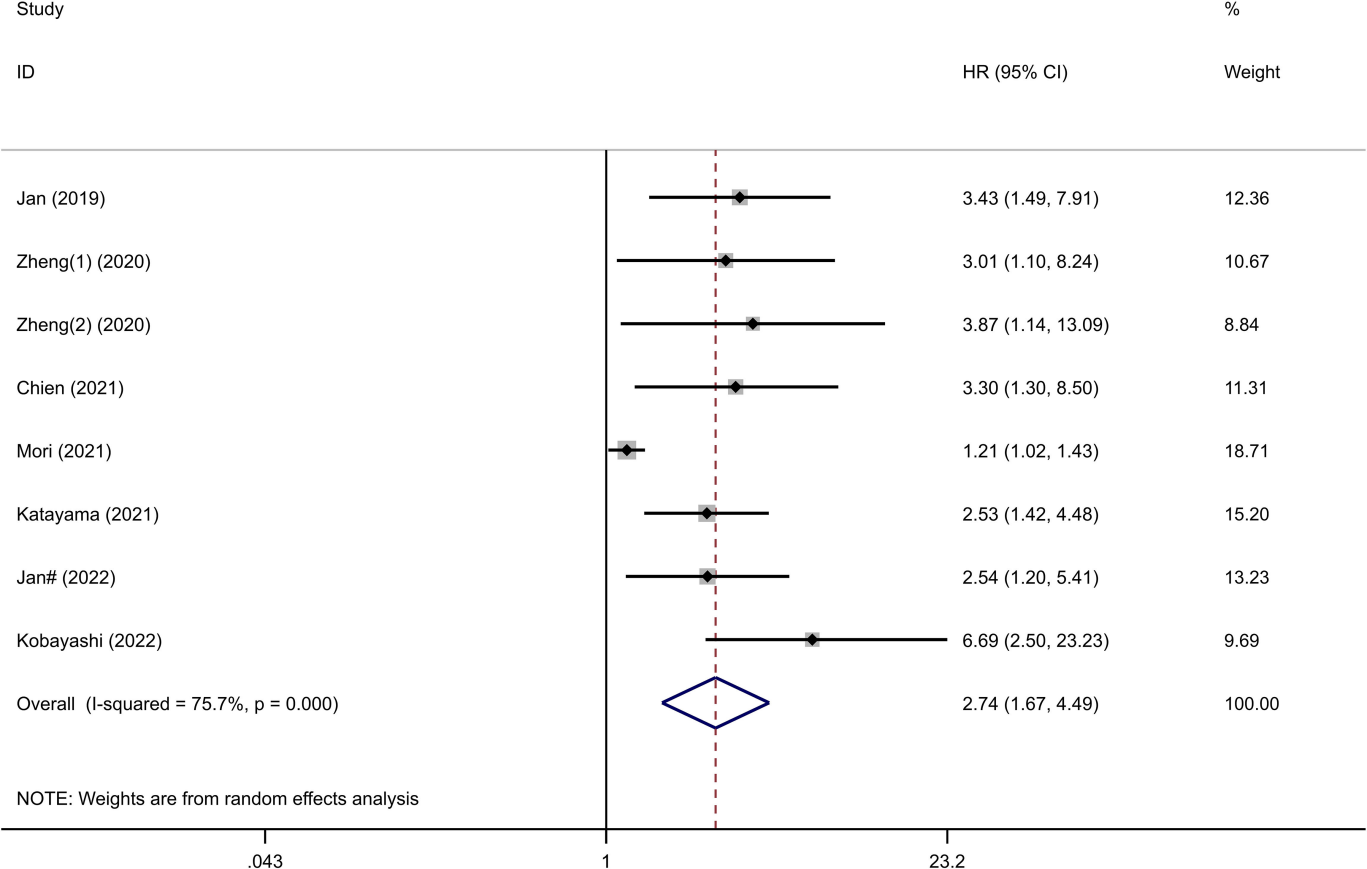
Forest plots showing the association between SII and CSS in UC.

The relationship between the SII and CSS was investigated in all seven studies involving 5317 patients. Results from their analyses suggested a significant association between elevated preoperative SII and poor CSS (HR=2.74, 95%CI 1.67-4.49, p<0.001), as depicted in **Figure 3**. Due to significant heterogeneity among the studies(I^2 ^= 75.7%, p<0.001), we conducted a subgroup analysis (**Supplementary Figure S2**). The results indicated that in all subgroups, high SII remained significantly associated with poor CSS (HR>1, 95%CI not overlapping 1), as depicted in **Table 3**.

**Table 3:** The subgroup analysis of the prognostic role of SII for CSS in UC.

Variables	No.of studies	Sample	HR (95% CI)	P	Heterogeneity		Model
					I^2^ (%)	P	
CSS							
Total	8	5317	2.74 (1.67-4.49)	<0.001	75.7	<0.001	Random
**Region**							Random
China	5	1724	3.09 (2.06-4.66)	<0.001	0	0.977	
No-China	3	3593	2.37 (1.02-5.51)	0.046	85.8	0.001	
**Sample**							Random
<300	3	628	4.18 (2.21-7.91)	<0.001	0	0.575	
≥300	5	4689	2.26 (1.32-3.85)	0.003	76.4	0.002	
**Cancer**							Random
UTUC	7	4200	2.83 (1.59-5.03)	<0.001	76.3	<0.001	
BC	1	1117	2.53 (1.42-4.49)	0.002	–	–	
**Cut-off**							Random
≤580	6	4792	2.62 (1.49-4.59)	0.001	79.5	<0.001	
>580	2	525	3.33 (1.53-7.25)	0.002	0	0.756	
**NOS**							Random
<9	3	1199	3.01 (1.86-4.86)	<0.001	0	0.852	
≥9	5	4118	2.63 (1.35-5.12)	0.004	79.3	0.001	

#### 3.3.3 Impact of the SII for RFS and PFS in urothelial carcinoma

**Figure 4:**
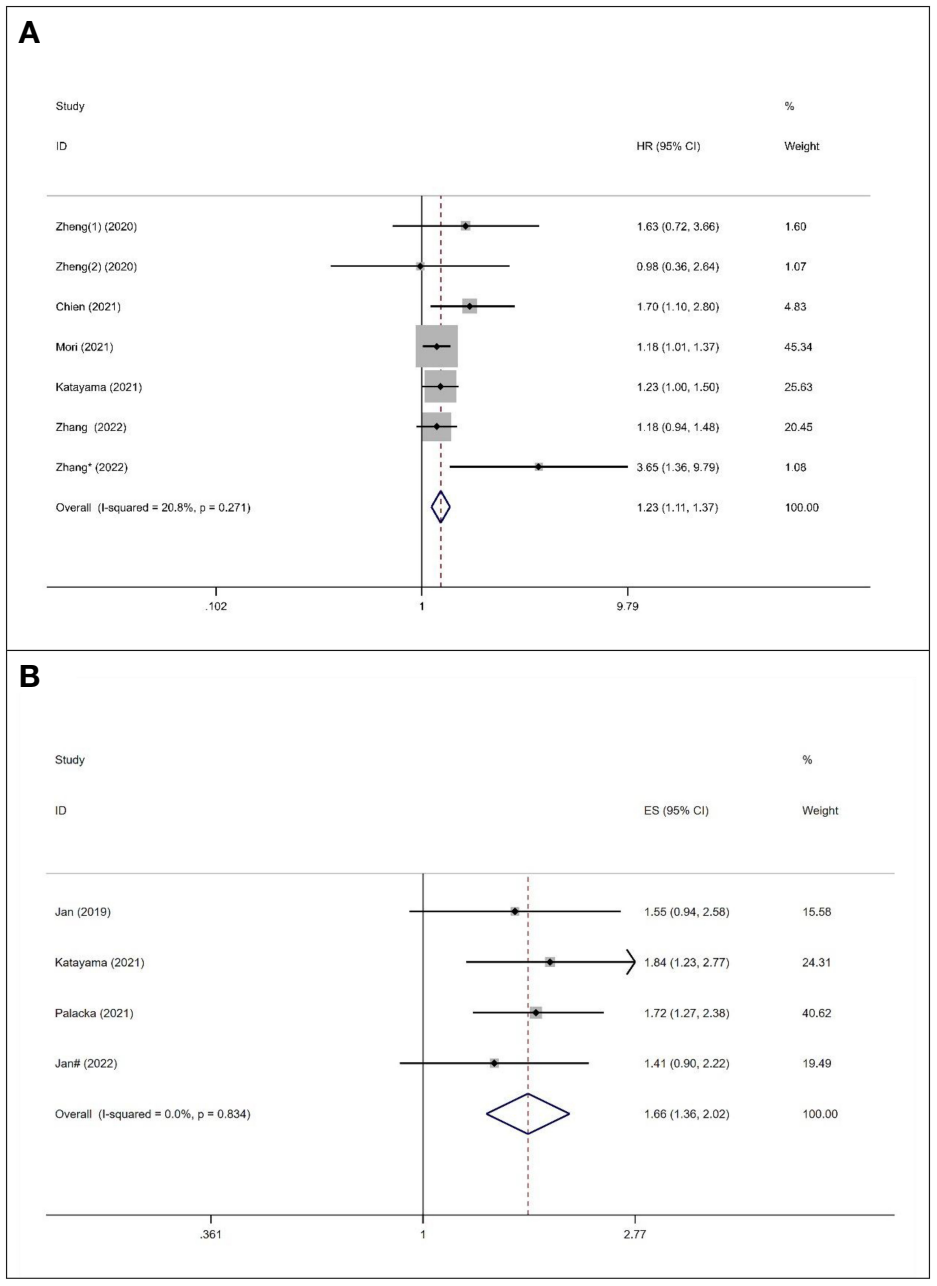
Forest plot of survival outcomes for UC. **(A)** RFS; **(B)** PFS.

Six studies, with a total of 5226 subjects, investigated the relationship between SII and RFS in UC. The analysis revealed a significant association between elevated preoperative SII and poor RFS (HR=1.23, 95%CI 1.11-1.37, p<0.001), as depicted in **Figure 4A**. No significant heterogeneity was observed among the studies (I^2 ^= 20.8%, p=0.270). A total of 4 studies, including 2121 patients, reported the prognostic value of preoperative SII on PFS in UC patients.

The combined data demonstrated that compared with a low SII, a high SII was significantly associated with poor PFS (HR=1.66, 95%CI 1.36-2.02, p<0.001), as shown in **Figure 4B**. No significant heterogeneity was observed among the studies (I^2 ^= 0%, p=0.834).

### 3.4 The Correlation Between SII and Clinical Pathological Factors in UC.

**Figure 5:**
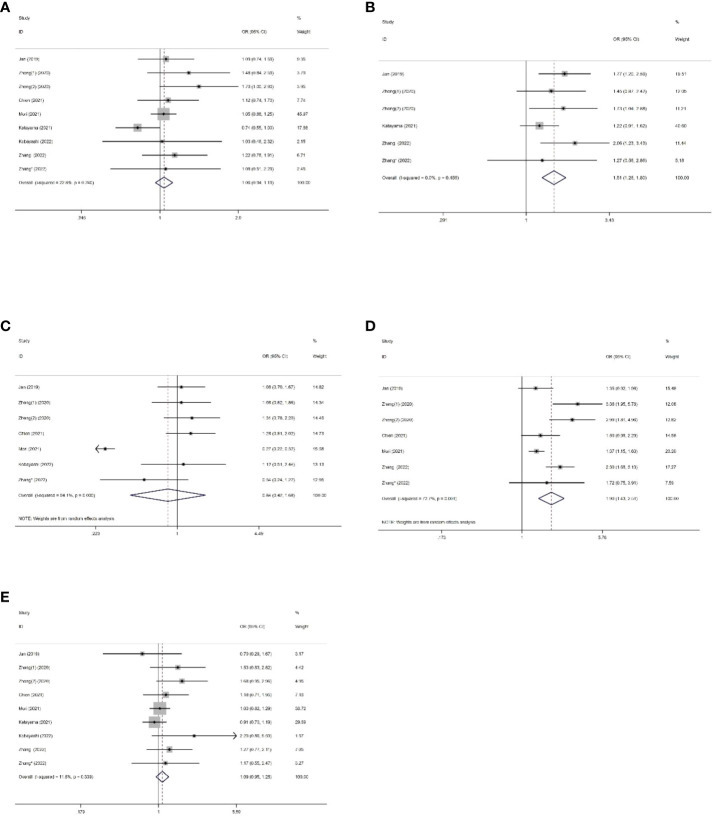
Forest plots of the association between SII and clinical pathological features of US. **(A)** Gender; **(B)** Tumour size; **(C)** Tumor location; **(D)** Pathologic T stage; **(E)** Tumor grade.

We collected data on the relationship between SII and various clinical pathological factors, including gender (male vs. female), tumor size (large vs. small), tumor location (renal pelvis vs. ureter), pathological T stage (>T2 vs. ≤T2), and tumor grade (high vs. low). A total of 8 studies, comprising 9 datasets, were included in the analysis. (**Figures 5A-E**, **Table 4**). The results demonstrated that high SII was significantly associated with large tumor size (OR=1.51, 95%CI 1.26-1.80, p<0.001) and advanced pathological T stage (OR=1.90, 95%CI 1.43-2.51, p<0.001) (**Figures 5B, D**, **Table 4**). However, no significant associations were observed between SII and gender (OR=1.06, 95%CI 0.94-1.91, p=0.0351), tumor location (OR=0.84, 95%CI 0.42-1.68, p=0.7967), and tumor grade (OR=1.09, 95%CI 0.95-1.25, p=0.227) (**Figures 5A, C, E**, **Table 4**).

**Table 4:** The correlation between SII and clinical pathological features in patients with UC.

Variables	No.of	Sample	OR( 95% CI)	P	Heterogeneity		Model
	studies				I^2^ (%)	P	
Gender(male vs female)	9	5753	1.06 (0.94-1.19)	0.0351	22.8	0.24	fixed
Tumour size(large vs small)	6	2901	1.51 (1.26-1.80)	<0.001	0	0.456	fixed
Tumor location(renal pelvis vs ureter )	7	3884	0.84 (0.42-1.68)	0.7967	94.1	<0.001	Random
Pathologic T stage(>T2 vs ≤T2)	7	4533	1.90 (1.43-2.51)	<0.001	72.7	0.001	Random
Tumor grade(high vs low)	9	5753	1.09 (0.95-1.25)	0.227	11.5	0.339	fixed

## 4 Publication bias

**Figure 6:**
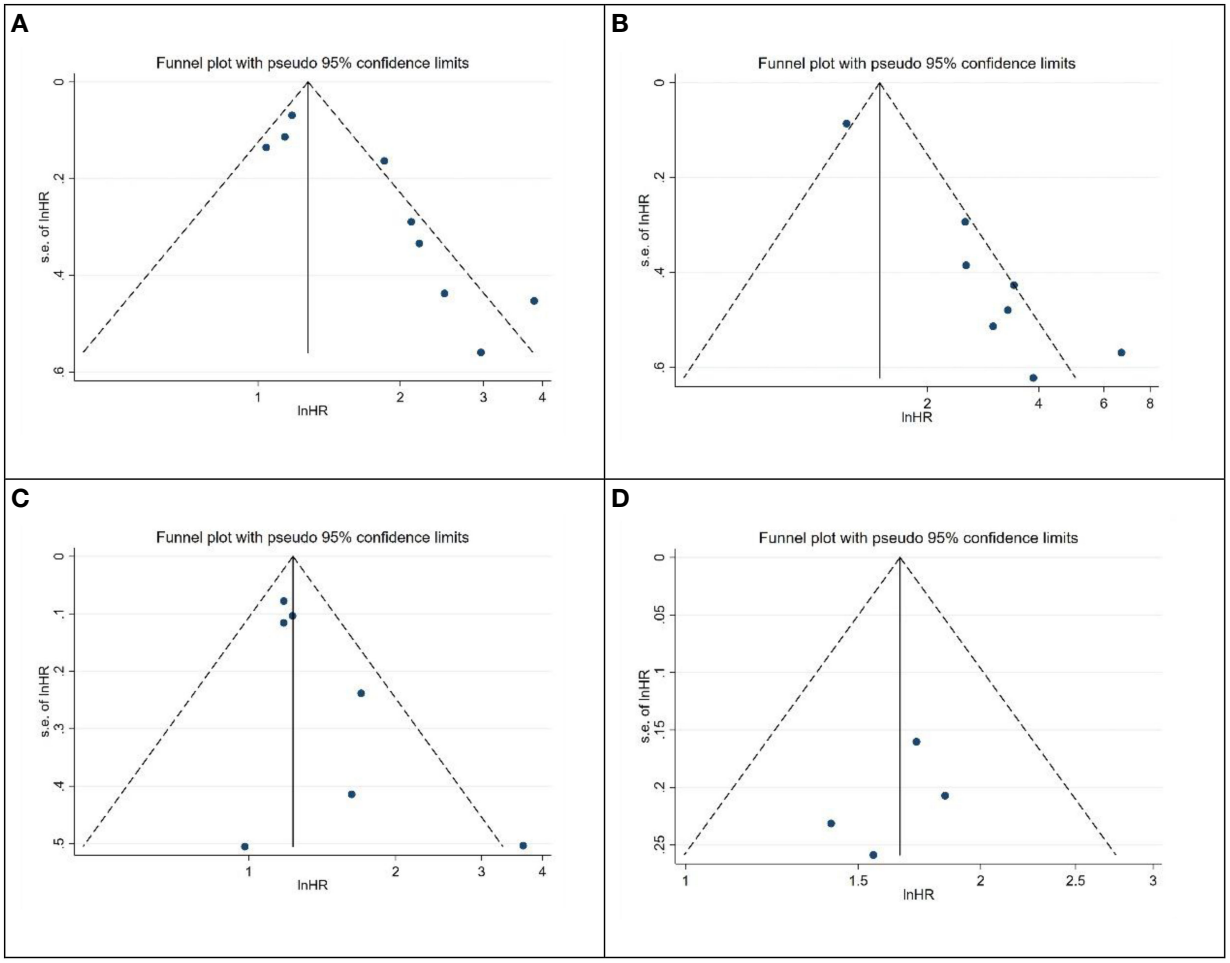
Publication bias assessment using funnel plots. **(A)** OS; **(B)** CSS; **(C)** RFS; **(D)** PFS.

**Figure 7:**
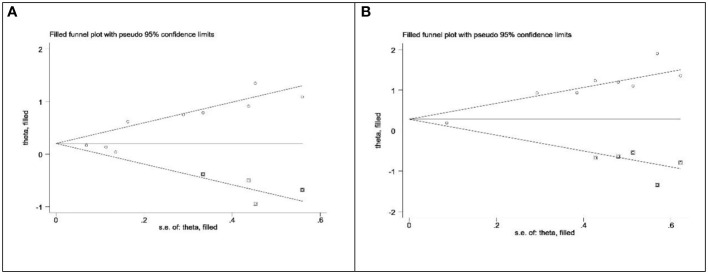
Duval and Tweedie’s nonparametric trim and fill method. **(A)** OS; **(B)** CSS.

The asymmetry of the funnel plot was observed (**Figures 6A-D**). We assessed publication bias using Begg’s test and Egger’s test for the included studies on prognostic value. The results of Begg’s test indicated no significant publication bias for all survival outcomes, including OS (p=0.348), CSS (p=0.563), RFS (p=0.368), and PFS (p=0.734). However, Egger’s test revealed significant publication bias for OS (p=0.005) and CSS (p<0.001). we employed the “trim and fill” method (**Figures 7A, B**). After filling in the potentially missing studies, the results suggested that the analysis results for OS and CSS might be unstable.

## 5 Sensitivity analysis

**Figure 8:**
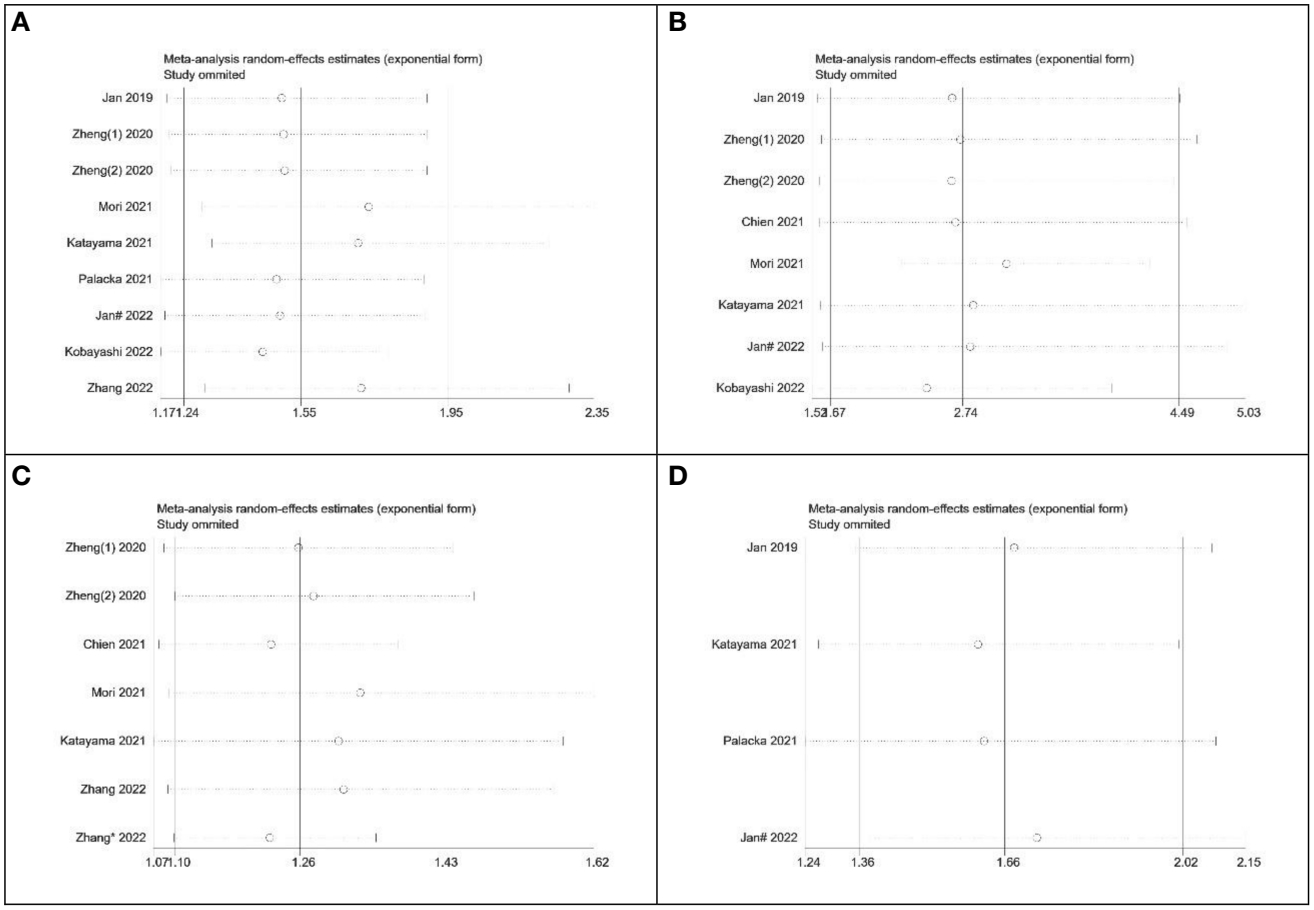
Sensitivity analysis of the relationship between SII and survival outcomes. **(A)** OS; **(B)** CSS; **(C)** RFS; **(D)** PFS.

To assess the robustness of the meta-analysis, a sensitivity analysis was conducted by systematically excluding one study at a time and evaluating the reliability of hazard ratios (HR) for overall survival (OS), cancer-specific survival (CSS), recurrence-free survival (RFS), and progression-free survival (PFS). The results indicated that the exclusion of any individual study did not significantly impact the overall conclusions (**Figures 8A-D**). This finding supports the relative stability and reliability of the meta-analysis results.

## 6 Discussion

Inflammation plays a critical role in the initiation and progression of cancer and is closely linked to various biological systems and systemic diseases (41). The systemic inflammatory response has been shown to promote cancer progression and contribute to different stages of tumor development, including initiation, invasion, angiogenesis, and metastasis (42). In recent years, the association between the SII and the prognosis of various cancer types has been extensively investigated, demonstrating its potential as a prognostic indicator, particularly for predicting survival outcomes in patients with malignant tumors (16, 17, 19–25, 27).

SII has been identified as a valuable prognostic indicator for numerous solid tumors, including hepatocellular carcinoma, pancreatic cancer, gallbladder cancer, bile duct cancer, gastric cancer, laryngeal cancer, endometrial cancer, non-small cell lung cancer, and bladder cancer, among others. In urological cancers, several studies have examined the prognostic value of SII, although the findings are not entirely consistent (26, 28, 43, 44). The biological principle of SII as a prognostic indicator is that it reflects the changes in the inflammatory state and immune function of the body. The inflammatory state can lead to an increase in platelets and neutrophils and a decrease in lymphocytes, thereby changing the value of SII. On the other hand, SII was calculated based on peripheral blood indicators (45). This means that all the necessary data for calculating SII can be obtained from routine blood tests, making it easily accessible and computationally convenient (9), without imposing additional medical burden on patients. Of course, there are other preoperative serum inflammatory biomarkers, such as NLR, PLR, and MLR, which have also been associated with outcomes in various cancer patients (46–49). Considering the multicellular components together, these inflammation-based biomarkers seem overly simplistic compared to SII, as they only consider two circulating immune cell types (46). In terms of comprehensive performance, SII comprehensively considers the number of different cell types, which can more accurately reflect the overall situation of inflammation and immune status and has better comprehensive performance. However, NLR, PLR, and MLR only reflect the ratio of specific cell types and cannot provide such comprehensive information. Several explanations have been proposed regarding the roles of neutrophils, platelets, and lymphocytes in cancer progression, highlighting the complex interplay between the immune system and tumor microenvironment (50–52). In conclusion, SII holds promise as a prognostic indicator in cancer patients, including those with urological cancers (26). However, further research is needed to establish consistent and reliable guidelines for its use in predicting prognosis and guiding treatment decisions for patients with urological cancers.

Platelets, neutrophils, and lymphocytes play important roles in the tumor microenvironment, and their interactions have complex effects on the development and progression of tumors. In recent years, more and more studies have shown that the interaction of these cells in the tumor microenvironment has an important impact on the invasion, metastasis, and prognosis of tumors. According to reports, platelets play a crucial role in multiple processes during cancer metastasis (53). The interaction between tumor cells and platelets is a prerequisite for successful hematogenous metastasis. When tumor cells enter the bloodstream, they immediately activate platelets, creating a loose microenvironment. Platelets provide protection to tumor cells against shear forces and attacks by natural killer (NK) cells (54), recruit bone marrow cells through the secretion of chemotactic factors, and facilitate the adherence of tumor cells and platelet emboli to the vascular wall. Subsequently, platelet-derived growth factors induce a mesenchymal-like phenotype in tumor cells and enhance the permeability of capillary endothelium, promoting the extravasation of tumor cells into distant organs. Finally, platelet-secreted growth factors stimulate tumor cell proliferation, leading to the formation of micro-metastatic foci.

Neutrophils are key effectors and regulators of the immune system. They can activate endothelial and parenchymal cells, facilitating the transfer of tumor cells in circulation. Neutrophils also play a significant role in the formation of neutrophil extracellular traps (NETs), which occur when neutrophils release their decondensed chromatin along with granule contents (55). Studies have found that central granulocytes can promote tumor angiogenesis, invasion, and metastasis of tumor cells by releasing pro-angiogenic factors, proteases, and cytokines (56). Second, neutrophils can also affect tumor immune escape and drug resistance by regulating the expression of cytokines and chemical factors in the tumor microenvironment (57). In addition, some studies have found that the number and activity of central granulocytes are closely related to the prognosis of tumors, and the high density of central granulocyte infiltration is related to the malignant degree and poor prognosis of tumors (58). In contrast, lymphocytes are the primary immune cell type and play a crucial role in eliminating tumor cells through immune surveillance (59, 60). A decrease in lymphocyte counts usually indicates the severity of the disease (61). Firstly, the role of lymphocytes in the tumor microenvironment mainly includes anti-tumor immune response and regulation of the tumor microenvironment. Lymphocytes play an anti-tumor role by recognizing and eliminating tumor cells. Among them, T cells and B cells are the two major lymphocyte subsets. T cells can directly kill tumor cells by recognizing tumor-specific antigens. B cells can produce antibodies and participate in the humoral immune response. Secondly, these effects are easily affected by external factors. For example, radiation can cause immunogenic oxidative stress and membrane damage in cancer cells, leading to the release of neoantigens and tumor antigens. In addition, radiotherapy adjusts the immunosuppressive tumor microenvironment by reducing immunosuppressive cytokines and polarizing macrophages toward an immune-stimulating phenotype (62).

In addition to direct anti-tumor effects, lymphocytes can also regulate the tumor microenvironment and inhibit tumor growth and spread by releasing cytokines such as interferon-γ (IFN-γ) and tumor necrosis factor (TNF). However, some studies have found that tumor cells can inhibit the activity of lymphocytes by releasing immunosuppressive factors such as transforming growth factor-β (TGF-β) and interleukin-10 (IL-10) in the tumor microenvironment, thereby escaping immune clearance. Studies have shown that lymphocytes can affect the expression of extracellular matrix components and proteases in the tumor microenvironment, thereby promoting tumor cell invasion and metastasis. In addition, lymphocytes can also affect tumor angiogenesis, epithelial-mesenchymal transition of tumor cells, and other processes, and further affect tumor metastasis and invasion (63).

In this meta-analysis, we included 10 studies (11 datasets) involving 6,333 patients to quantitatively investigate the role of preoperative SII as a prognostic indicator in UC patients and its potential value in clinical pathological features. The results showed that a higher preoperative SII was significantly associated with poorer OS, CSS, RFS, and PFS in UC patients. Subgroup analyses also indicated that a higher SII was an important prognostic factor for poor OS and CSS in UC patients, considering factors such as race, sample size, and treatment modality. Moreover, the pooled data on clinical pathological features revealed that an elevated SII in UC patients was correlated with larger tumor size and later pathological T stage. In conclusion, a high preoperative SII can serve as an independent prognostic marker for poor survival in UC patients and holds important clinical utility. Additionally, due to the significant association between SII and adverse clinical pathological features, monitoring preoperative SII may aid in the early detection of disease progression, enabling timely intervention and treatment. To the best of our knowledge, this is the first meta-analysis to investigate the role and clinical pathological significance of preoperative SII in predicting the prognosis of UC patients. Therefore, future studies should further explore the clinical application of SII in different types of tumors to verify its practicality in prognosis assessment and disease progression monitoring. Moreover, by exploring the standardized cut-off value of SII and conducting large-scale prospective studies, its limitations can be better addressed and more specific recommendations can be provided for future studies.

It is important to note that there are several limitations to this study. Firstly, all the studies included in this meta-analysis were retrospective, which may introduce selection bias. The assessment using the “trim and fill” method suggested the presence of publication bias. Secondly, there were variations in the cut-off or threshold values for SII among the studies, which may contribute to heterogeneity. Thirdly, the heterogeneity of OS and CSS in this study may be due to other unknown factors. Fourth, some studies had relatively small sample sizes, which may limit the reliability of this study, and future population-based studies are necessary. Therefore, due to the aforementioned limitations, further large-scale prospective studies are needed to confirm our findings. In addition to these limitations, the prediction of prognosis in UC is an area of constant innovation. Liquid biopsy is a family of techniques designed to obtain cancer information from its circulating by-products (e.g., circulating tumor cells, circulating tumor DNA), facilitating biomarker detection. More advanced and rational treatment models (e.g., concomitant immunotherapy, metastasis-directed radiotherapy, and multimodal therapy) have been introduced through biomarker drive (64). Finally, more accurate molecular biomarkers may come from gene expression profiles, which we need to explore further.

## 7 Conclusion

Our systematic review and meta-analysis suggest that SII can be used as a survival and prognostic predictor in patients with urothelial carcinoma, which has important clinical significance. However, prospective studies are needed to further verify its reliability and accuracy. This finding is of great significance for the treatment and monitoring of patients with urothelial carcinoma and can provide an important basis for individualized treatment and prognosis evaluation. Therefore, future studies should focus on the clinical application of SII in patients with urothelial carcinoma to verify its reliability in clinical practice and provide more accurate guidance for the treatment of patients. In the future, we believe that more predictive biomarkers will be developed to provide more accurate prognostic information and help with treatment for UC patients.

## Data availability statement

The datasets presented in this study can be found in online repositories. The names of the repository/repositories and accession number(s) can be found in the article/**Supplementary Material**.

## Author contributions

YW: Conceptualization, Data curation, Formal analysis, Methodology, Software, Supervision, Validation, Visualization, Writing – original draft, Writing – review & editing. GL: Conceptualization, Data curation, Investigation, Methodology, Supervision, Validation, Writing – review & editing. XH: Data curation, Investigation, Methodology, Supervision, Validation, Writing – review & editing.

## References

[1] Babjuk M Böhle A Burger M Capoun O Cohen D Compérat EM . Eau guidelines on non-muscle-invasive urothelial carcinoma of the bladder: update 2016. Eur Urol. (2017) 71:447–61. doi: 10.1016/j.eururo.2016.05.041 27324428

[2] Gakis G Witjes JA Compérat E Cowan NC De Santis M Lebret T . Eau guidelines on primary urethral carcinoma. Eur Urol. (2013) 64:823–30. doi: 10.1016/j.eururo.2013.03.044 23582479

[3] Rouprêt M Seisen T Birtle AJ Capoun O Compérat EM Dominguez-Escrig JL . European association of urology guidelines on upper urinary tract urothelial carcinoma: 2023 update. Eur Urol. (2023) 84:49–64. doi: 10.1016/j.eururo.2023.03.013 36967359

[4] Flaig TW Spiess PE Abern M Agarwal N Bangs R Boorjian SA . Nccn guidelines® Insights: bladder cancer, version 2.2022. J Natl Compr Canc Netw. (2022) 20:866–78. doi: 10.6004/jnccn.2022.0041 35948037

[5] Soria F Shariat SF Lerner SP Fritsche HM Rink M Kassouf W . Epidemiology, diagnosis, preoperative evaluation and prognostic assessment of upper-tract urothelial carcinoma (Utuc). World J Urol. (2017) 35:379–87. doi: 10.1007/s00345-016-1928-x 27604375

[6] Wang Q Zhang T Wu J Wen J Tao D Wan T . Prognosis and risk factors of patients with upper urinary tract urothelial carcinoma and postoperative recurrence of bladder cancer in Central China. BMC Urol. (2019) 19:24. doi: 10.1186/s12894-019-0457-5 30999871 PMC6471846

[7] Giudici N Bonne F Blarer J Minoli M Krentel F Seiler R . Characteristics of upper urinary tract urothelial carcinoma in the context of bladder cancer: A narrative review. Transl Androl Urol. (2021) 10:4036–50. doi: 10.21037/tau-20-1472 PMC8575564 34804846

[8] Tomiyama E Fujita K Hashimoto M Adomi S Kawashima A Minami T . Comparison of molecular profiles of upper tract urothelial carcinoma vs. Urinary bladder cancer in the era of targeted therapy: A narrative review. Transl Androl Urol. (2022) 11:1747–61. doi: 10.21037/tau-22-457 PMC9827402 36632153

[9] Ravindranathan D Master VA Bilen MA . Inflammatory markers in cancer immunotherapy. Biol (Basel). (2021) 10(4):325. doi: 10.3390/biology10040325 PMC8069970 33924623

[10] Di Raimondo C Lombardo P Tesei C Esposito F Meconi F Secchi R . Role of neutrophil-to-lymphocyte ratio (Nlr) in patients with mycosis fungoides. Diagnostics (Basel). (2023) 13(11):1979. doi: 10.3390/diagnostics13111979 37296831 PMC10252222

[11] Zhu Y Li M Bo C Liu X Zhang J Li Z . Prognostic significance of the lymphocyte-to-monocyte ratio and the tumor-infiltrating lymphocyte to tumor-associated macrophage ratio in patients with stage T3n0m0 esophageal squamous cell carcinoma. Cancer Immunol Immunother. (2017) 66:343–54. doi: 10.1007/s00262-016-1931-5 PMC11029213 27915370

[12] Duque-Santana V López-Campos F Martin-Martin M Valero M Zafra-Martín J Couñago F . Neutrophil-to-lymphocyte ratio and platelet-to-lymphocyte ratio as prognostic factors in locally advanced rectal cancer. Oncology. (2023) 101:349–57. doi: 10.1159/000526450 36273439

[13] Yang C Hu BW Tang F Zhang Q Quan W Wang J . Prognostic value of systemic immune-inflammation index (Sii) in patients with glioblastoma: A comprehensive study based on meta-analysis and retrospective single-center analysis. J Clin Med. (2022) 11(24):7514. doi: 10.3390/jcm11247514 36556130 PMC9787672

[14] Ding P Lv J Sun C Chen S Yang P Tian Y . Combined systemic inflammatory immunity index and prognostic nutritional index scores as a screening marker for sarcopenia in patients with locally advanced gastric cancer. Front Nutr. (2022) 9:981533. doi: 10.3389/fnut.2022.981533 36046129 PMC9421237

[15] Wang B Huang Y Lin T . Prognostic impact of elevated pre-treatment systemic immune-inflammation index (Sii) in hepatocellular carcinoma: A meta-analysis. Med (Baltimore). (2020) 99:e18571. doi: 10.1097/md.0000000000018571 PMC6946450 31895801

[16] Hu B Yang XR Xu Y Sun YF Sun C Guo W . Systemic immune-inflammation index predicts prognosis of patients after curative resection for hepatocellular carcinoma. Clin Cancer Res. (2014) 20:6212–22. doi: 10.1158/1078-0432.Ccr-14-0442 25271081

[17] Chen Q Ren S Cui S Huang J Wang D Li B . Prognostic and recurrent significance of sii in patients with pancreatic head cancer undergoing pancreaticoduodenectomy. Front Oncol. (2023) 13:1122811. doi: 10.3389/fonc.2023.1122811 37284203 PMC10240062

[18] Rovesti G Leone F Brandi G Fornaro L Scartozzi M Niger M . Prognostic role of a new index tested in european and korean advanced biliary tract cancer patients: the pecs index. J Gastrointest Cancer. (2022) 53:289–98. doi: 10.1007/s12029-021-00596-z 33544375

[19] Terasaki F Sugiura T Okamura Y Ito T Yamamoto Y Ashida R . Systemic immune-inflammation index as a prognostic marker for distal cholangiocarcinoma. Surg Today. (2021) 51:1602–9. doi: 10.1007/s00595-021-02312-7 34142236

[20] Uzunoglu H Kaya S . Does systemic immune inflammation index have predictive value in gastric cancer prognosis? North Clin Istanb. (2023) 10:24–32. doi: 10.14744/nci.2021.71324 36910431 PMC9996656

[21] Atasever Akkas E Yucel B . Prognostic Value of Systemic ımmune ınflammation ındex in Patients with Laryngeal Cancer. Eur Arch Otorhinolaryngol. (2021) 278:1945–55. doi: 10.1007/s00405-021-06798-2 33837464

[22] Huang Y Chen Y Zhu Y Wu Q Yao C Xia H . Postoperative systemic immune-inflammation index (Sii): A superior prognostic factor of endometrial cancer. Front Surg. (2021) 8:704235. doi: 10.3389/fsurg.2021.704235 34746222 PMC8568766

[23] Mazzella A Maiolino E Maisonneuve P Loi M Alifano M . Systemic inflammation and lung cancer: is it a real paradigm? Prognostic value of inflammatory indexes in patients with resected non-small-cell lung cancer. Cancers (Basel). (2023) 15(6):1854. doi: 10.3390/cancers15061854 36980740 PMC10046843

[24] Yilmaz H Cinar NB Avci IE Telli E Uslubas AK Teke K . The systemic inflammation response index: an independent predictive factor for survival outcomes of bladder cancer stronger than other inflammatory markers. Urol Oncol. (2023) 41:256.e251–256.e258. doi: 10.1016/j.urolonc.2022.11.011 36577568

[25] Fornarini G Rebuzzi SE Banna GL Calabrò F Scandurra G De Giorgi U . Immune-inflammatory biomarkers as prognostic factors for immunotherapy in pretreated advanced urinary tract cancer patients: an analysis of the italian saul cohort. ESMO Open. (2021) 6:100118. doi: 10.1016/j.esmoop.2021.100118 33984678 PMC8134706

[26] Wang Q Zhu SR Huang XP Liu XQ Liu JB Tian G . Prognostic value of systemic immune-inflammation index in patients with urinary system cancers: A meta-analysis. Eur Rev Med Pharmacol Sci. (2021) 25:1302–10. doi: 10.26355/eurrev_202102_24834 33629300

[27] Gu Y Fu Y Pan X Zhou Y Ji C Zhao T . Prognostic value of systemic immune-inflammation index in non-metastatic clear cell renal cell carcinoma with tumor thrombus. Front Oncol. (2023) 13:1117595. doi: 10.3389/fonc.2023.1117595 36776325 PMC9909392

[28] Zhang B Xu T . Prognostic significance of pretreatment systemic immune-inflammation index in patients with prostate cancer: A meta-analysis. World J Surg Oncol. (2023) 21:2. doi: 10.1186/s12957-022-02878-7 36600256 PMC9814343

[29] Page MJ Mckenzie JE Bossuyt PM Boutron I Hoffmann TC Mulrow CD . The prisma 2020 statement: an updated guideline for reporting systematic reviews. Bmj. (2021) 372:n71. doi: 10.1136/bmj.n71 33782057 PMC8005924

[30] Stang A . Critical evaluation of the newcastle-ottawa scale for the assessment of the quality of nonrandomized studies in meta-analyses. Eur J Epidemiol. (2010) 25:603–5. doi: 10.1007/s10654-010-9491-z 20652370

[31] Jan H-C Yang W-H Ou C-H . Combination of the preoperative systemic immune-inflammation index and monocyte-lymphocyte ratio as a novel prognostic factor in patients with upper-tract urothelial carcinoma. Ann Surg Oncol. (2019) 26:669–84. doi: 10.1245/s10434-018-6942-3 30374917

[32] Zheng Y Yu D Yu Z Zhao D Chen Y Chen W . Association of preoperative systemic immune-inflammation index and prognostic nutritional index with survival in patients with upper tract urothelial carcinoma. J Cancer. (2020) 11:5665–77. doi: 10.7150/jca.44915 PMC7477451 32913461

[33] Chien TM Li CC Lu YM Chou YH Chang HW Wu WJ . The predictive value of systemic immune-inflammation index on bladder recurrence on upper tract urothelial carcinoma outcomes after radical nephroureterectomy. J Clin Med. (2021) 10(22):5273. doi: 10.3390/jcm10225273 PMC8623909 34830555

[34] Mori K Resch I Miura N Laukhtina E Schuettfort VM Pradere B . Prognostic role of the systemic immune-inflammation index in upper tract urothelial carcinoma treated with radical nephroureterectomy: results from a large multicenter international collaboration. Cancer Immunol Immunotherapy. (2021) 70:2641–50. doi: 10.1007/s00262-021-02884-w PMC8360829 33591412

[35] Katayama S Mori K Pradere B Laukhtina E Schuettfort VM Quhal F . Prognostic value of the systemic immune-inflammation index in non-muscle invasive bladder cancer. World J Urol. (2021) 39:4355–61. doi: 10.1007/s00345-021-03740-3 PMC8602174 34143284

[36] Palacka P Slopovsky J Obertova J Chovanec M Rejlekova K Sycova-Mila Z . Survival prediction by baseline systemic immune-inflammation index (Sii) and its changes during first-line platinum-based treatment in a caucasian population of patients with metastatic urothelial carcinoma (Muc). Anticancer Res. (2021) 41:5749–59. doi: 10.21873/anticanres.15391 34732448

[37] Jan H-C Wu K-Y Tai T-Y Weng H-Y Yang W-H Ou C-H . The systemic immune-inflammation index (Sii) increases the prognostic significance of lymphovascular invasion in upper tract urothelial carcinoma after radical nephroureterectomy. Cancer Manage Res. (2022) 14:3139–49. doi: 10.2147/CMAR.S378768 PMC9651009 36386553

[38] Kobayashi S Ito M Takemura K Suzuki H Yonese I Koga F . Preoperative models incorporating the systemic immune-inflammation index for predicting prognosis and muscle invasion in patients with non-metastatic upper tract urothelial carcinoma. Int J Clin Oncol. (2022) 27:574–84. doi: 10.1007/s10147-021-02088-3 34860315

[39] Zhang S Du J Zhong X Tan P Xu H Zhang J . The prognostic value of the systemic immune-inflammation index for patients with bladder cancer after radical cystectomy. Front Immunol. (2022) 13:1072433. doi: 10.3389/fimmu.2022.1072433 36524107 PMC9744948

[40] Zhang XW Gao Y Xu H Liu Y Hu XZ Lu GJ . Preoperative systemic immune-inflammation index to evaluate intravesical recurrence of upper urinary tract urothelial carcinoma treated with radical nephroureterectomy. J Modern Urol. (2022) 27:30–4. doi: 10.3696/j.issn.1009-8291.2022.01.006

[41] Coussens LM Werb Z . Inflammation and cancer. Nature. (2002) 420:860–7. doi: 10.1038/nature01322 PMC2803035 12490959

[42] Hanahan D Weinberg RA . Hallmarks of cancer: the next generation. Cell. (2011) 144:646–74. doi: 10.1016/j.cell.2011.02.013 21376230

[43] Jin M Yuan S Yuan Y Yi L . Prognostic and clinicopathological significance of the systemic immune-inflammation index in patients with renal cell carcinoma: A meta-analysis. Front Oncol. (2021) 11:735803. doi: 10.3389/fonc.2021.735803 34950577 PMC8689141

[44] Cao W Shao Y Zou S Wang N Wang J . Prognostic significance of systemic immune-inflammation index in patients with bladder cancer: A systematic review and meta-analysis. Med (Baltimore). (2022) 101:e30380. doi: 10.1097/md.0000000000030380 PMC10980366 36086786

[45] Hirahara N Tajima Y Matsubara T Fujii Y Kaji S Kawabata Y . Systemic immune-inflammation index predicts overall survival in patients with gastric cancer: A propensity score-matched analysis. J Gastrointest Surg. (2021) 25:1124–33. doi: 10.1007/s11605-020-04710-7 32607856

[46] Kosidło JW Wolszczak-Biedrzycka B Matowicka-Karna J Dymicka-Piekarska V Dorf J . Clinical significance and diagnostic utility of nlr, lmr, plr and sii in the course of covid-19: A literature review. J Inflammation Res. (2023) 16:539–62. doi: 10.2147/jir.S395331 PMC9930576 36818192

[47] Li P Li H Ding S Zhou J . Nlr, plr, lmr and mwr as diagnostic and prognostic markers for laryngeal carcinoma. Am J Transl Res. (2022) 14:3017–27. PMC9185085 35702077

[48] Gambardella C Mongardini FM Paolicelli M Bentivoglio D Cozzolino G Ruggiero R . Role of inflammatory biomarkers (Nlr, lmr, plr) in the prognostication of Malignancy in indeterminate thyroid nodules. Int J Mol Sci. (2023) 24(7):6466. doi: 10.3390/ijms24076466 37047439 PMC10094849

[49] Hu C Bai Y Li J Zhang G Yang L Bi C . Prognostic value of systemic inflammatory factors nlr, lmr, plr and ldh in penile cancer. BMC Urol. (2020) 20:57. doi: 10.1186/s12894-020-00628-z 32460817 PMC7251912

[50] Gonzalez H Hagerling C Werb Z . Roles of the immune system in cancer: from tumor initiation to metastatic progression. Genes Dev. (2018) 32:1267–84. doi: 10.1101/gad.314617.118 PMC6169832 30275043

[51] Adam JK Odhav B Bhoola KD . Immune responses in cancer. Pharmacol Ther. (2003) 99:113–32. doi: 10.1016/s0163-7258(03)00056-1 12804702

[52] Taniguchi K Karin M . Nf-Κb, inflammation, immunity and cancer: coming of age. Nat Rev Immunol. (2018) 18:309–24. doi: 10.1038/nri.2017.142 29379212

[53] Gaertner F Massberg S . Patrolling the vascular borders: platelets in immunity to infection and cancer. Nat Rev Immunol. (2019) 19:747–60. doi: 10.1038/s41577-019-0202-z 31409920

[54] Maurer S Ferrari De Andrade L . Nk cell interaction with platelets and myeloid cells in the tumor milieu. Front Immunol. (2020) 11:608849. doi: 10.3389/fimmu.2020.608849 33424862 PMC7785787

[55] Kolman JP Pagerols Raluy L Müller I Nikolaev VO Trochimiuk M Appl B . Net release of long-term surviving neutrophils. Front Immunol. (2022) 13:815412. doi: 10.3389/fimmu.2022.815412 35242132 PMC8887621

[56] Mao X Xu J Wang W Liang C Hua J Liu J . Crosstalk between cancer-associated fibroblasts and immune cells in the tumor microenvironment: new findings and future perspectives. Mol Cancer. (2021) 20:131. doi: 10.1186/s12943-021-01428-1 34635121 PMC8504100

[57] Tie Y Tang F Wei YQ Wei XW . Immunosuppressive cells in cancer: mechanisms and potential therapeutic targets. J Hematol Oncol. (2022) 15:61. doi: 10.1186/s13045-022-01282-8 35585567 PMC9118588

[58] Rubenich DS De Souza PO Omizzollo N Aubin MR Basso PJ Silva LM . Tumor-neutrophil crosstalk promotes in vitro and in vivo glioblastoma progression. Front Immunol. (2023) 14:1183465. doi: 10.3389/fimmu.2023.1183465 37292196 PMC10244780

[59] Quigley DA Kristensen V . Predicting prognosis and therapeutic response from interactions between lymphocytes and tumor cells. Mol Oncol. (2015) 9:2054–62. doi: 10.1016/j.molonc.2015.10.003 PMC5528725 26607741

[60] Man YG Stojadinovic A Mason J Avital I Bilchik A Bruecher B . Tumor-infiltrating immune cells promoting tumor invasion and metastasis: existing theories. J Cancer. (2013) 4:84–95. doi: 10.7150/jca.5482 23386907 PMC3564249

[61] Grisaru-Tal S Dulberg S Beck L Zhang C Itan M Hediyeh-Zadeh S . Metastasis-entrained eosinophils enhance lymphocyte-mediated antitumor immunity. Cancer Res. (2021) 81:5555–71. doi: 10.1158/0008-5472.Can-21-0839 34429328

[62] Hinshaw DC Shevde LA . The tumor microenvironment innately modulates cancer progression. Cancer Res. (2019) 79:4557–66. doi: 10.1158/0008-5472.Can-18-3962 PMC6744958 31350295

[63] Chen YF Shao GC Li J Yang AQ Li J Ye XS . O-glcnacylation of blimp-1 in lymphocytes inhibits its transcriptional function and is associated with migration and invasion of breast cancer cells. Mol Cancer Res. (2022) 20:650–60. doi: 10.1158/1541-7786.Mcr-21-0405 34907035

[64] Pezzicoli G Salonne F Musci V Ciciriello F Tommasi S Lacalamita R . Concomitant immunotherapy and metastasis-directed radiotherapy in upper tract urothelial carcinoma: A biomarker-driven, original, case-based proof-of-concept study. J Clin Med. (2023) 12(24):7761. doi: 10.3390/jcm12247761 38137830 PMC10744017

