# Supplementary material for: Prognostic and clinical pathological significance of the systemic immune-inflammation index in urothelial carcinoma: a systematic review and meta-analysis
Source: Front Oncol. 2024 Mar 26;14:1322897. doi: 10.3389/fonc.2024.1322897 (PMC11002112; doi:10.3389/fonc.2024.1322897)
Supplement: Supplementary file 1 [file DataSheet_1.docx]

Supplementary Material

**Table S1.** Database search strategy.

| Databases | Search Strategy | Results |
| --- | --- | --- |
| PubMed | (("systemic immune-inflammation index") OR ("SII")) AND ((((((("urothelial carcinoma") OR ("bladder carcinoma")) OR ("bladder cancer")) OR ("upper tract urothelial cancer")) OR ("upper tract urothelial carcinoma")) OR ("urethral carcinoma")) OR ("urethral cancer"))[All Fields] | 43 |
| Embase | ('systemic immune-inflammation index':ab,ti OR 'sii':ab,ti) AND ('urothelial carcinoma':ab,ti OR 'bladder carcinoma':ab,ti OR 'bladder cancer':ab,ti OR 'upper tract urothelial cancer':ab,ti OR 'upper tract urothelial carcinoma':ab,ti OR 'urethral carcinoma':ab,ti OR 'urethral cancer':ab,ti) | 57 |
| Web of science | ((TS=("systemic immune-inflammation index")) OR TS=("SII")) AND (((((((TS=("urothelial carcinoma")) OR TS=("bladder carcinoma")) OR TS=("bladder cancer")) OR TS=("upper tract urothelial cancer")) OR TS=("upper tract urothelial carcinoma")) OR TS=("urethral carcinoma")) OR TS=("urethral cancer")) | 55 |
| Cochrane Library | (("systemic immune-inflammation index"):ti,ab,kw OR ("SII"):ti,ab,kw) AND (("urothelial carcinoma"):ti,ab,kw OR ("bladder carcinoma"):ti,ab,kw OR ("bladder cancer"):ti,ab,kw OR ("upper tract urothelial cancer"):ti,ab,kw OR ("upper tract urothelial carcinoma"):ti,ab,kw OR ("urethral carcinoma"):ti,ab,kw OR ("urethral cancer"):ti,ab,kw) | 2 |
| CNKI | [(主题=全身免疫炎症指数) OR (主题=SII)](http://kns-cnki-net-443.webvpn.bjmu.tsg211.com/kns8/AdvSearch?id=1&dbcode=CFLS&searchtype=gradeSearch&ishistory=1" \o "(主题=全身免疫炎症指数) OR (主题=SII)" \t "http://kns-cnki-net-443.webvpn.bjmu.tsg211.com/kns8/manage/_blank) AND [(主题=尿路上皮癌) OR (主题=上尿路尿路上皮癌) OR (主题=膀胱癌) OR (主题=尿道癌)](http://kns-cnki-net-443.webvpn.bjmu.tsg211.com/kns8/AdvSearch?id=2&dbcode=CFLS&searchtype=gradeSearch&ishistory=1" \o "(主题=尿路上皮癌) OR (主题=上尿路尿路上皮癌) OR (主题=膀胱癌) OR (主题=尿道癌)" \t "http://kns-cnki-net-443.webvpn.bjmu.tsg211.com/kns8/manage/_blank) | 17 |
| ALL |  | 174 |

**Table S2.** Newcastle-Ottawa quality assessments scale.

| **(A)**  **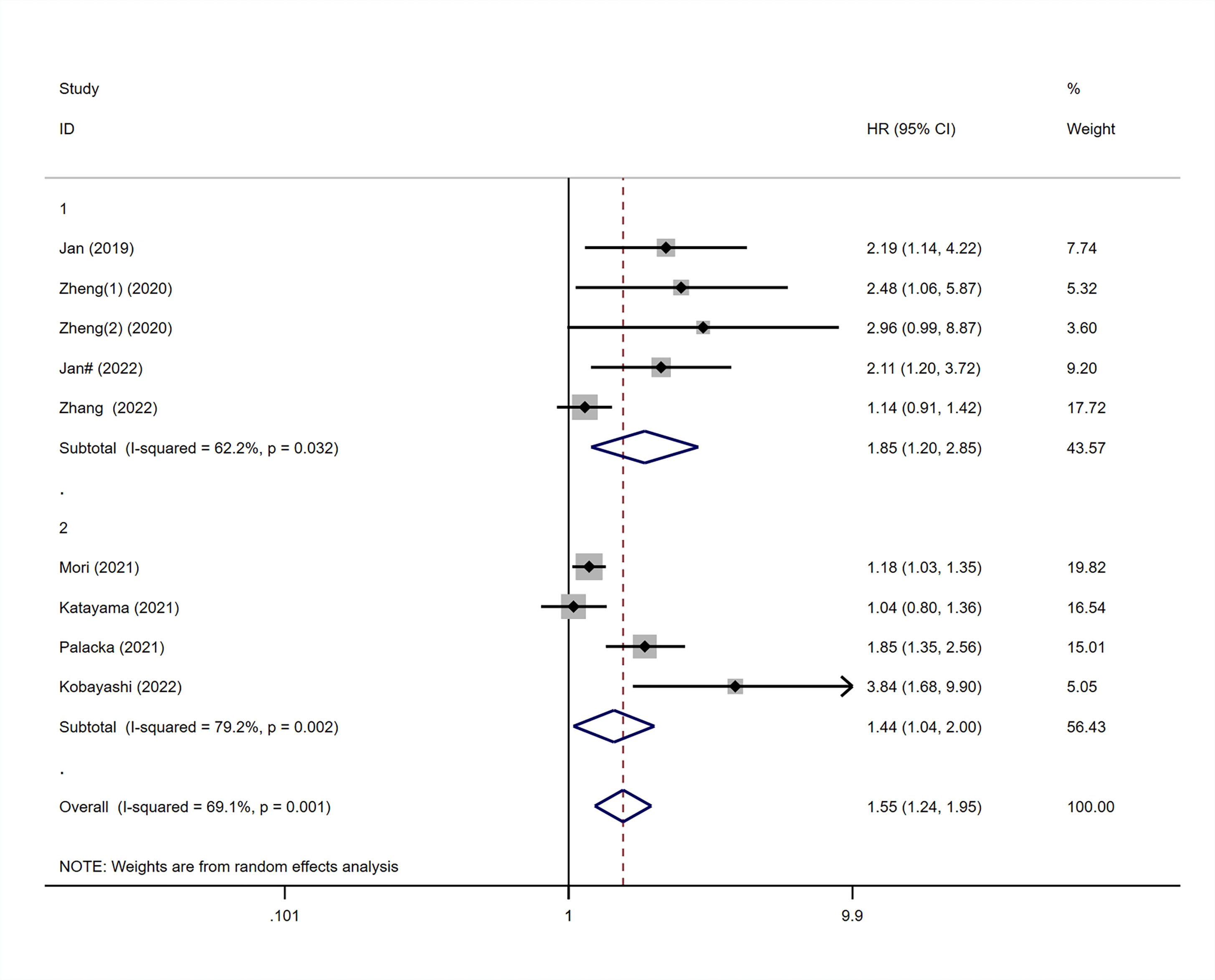** | **(B)**  **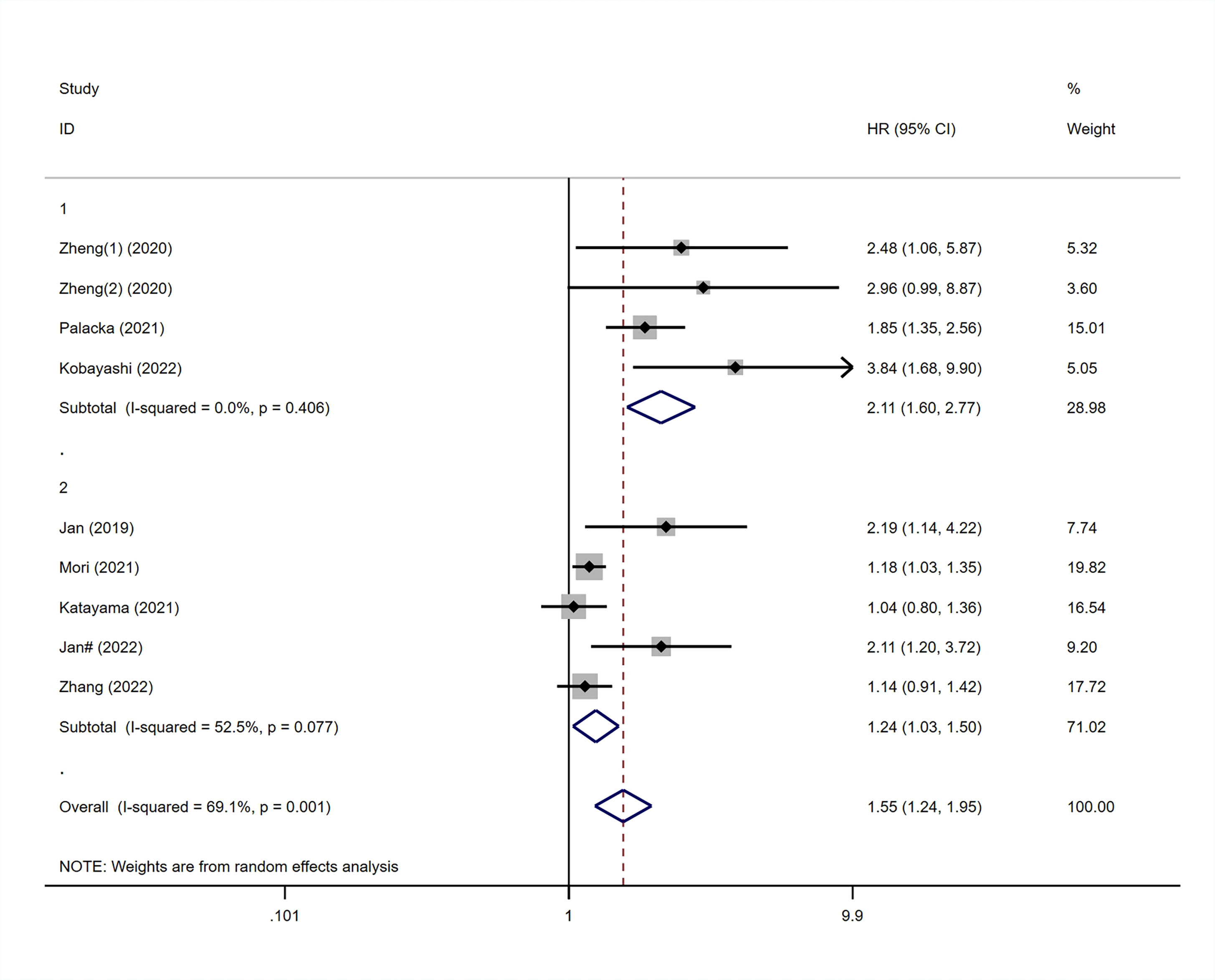** |
| --- | --- |
| **(C)**  **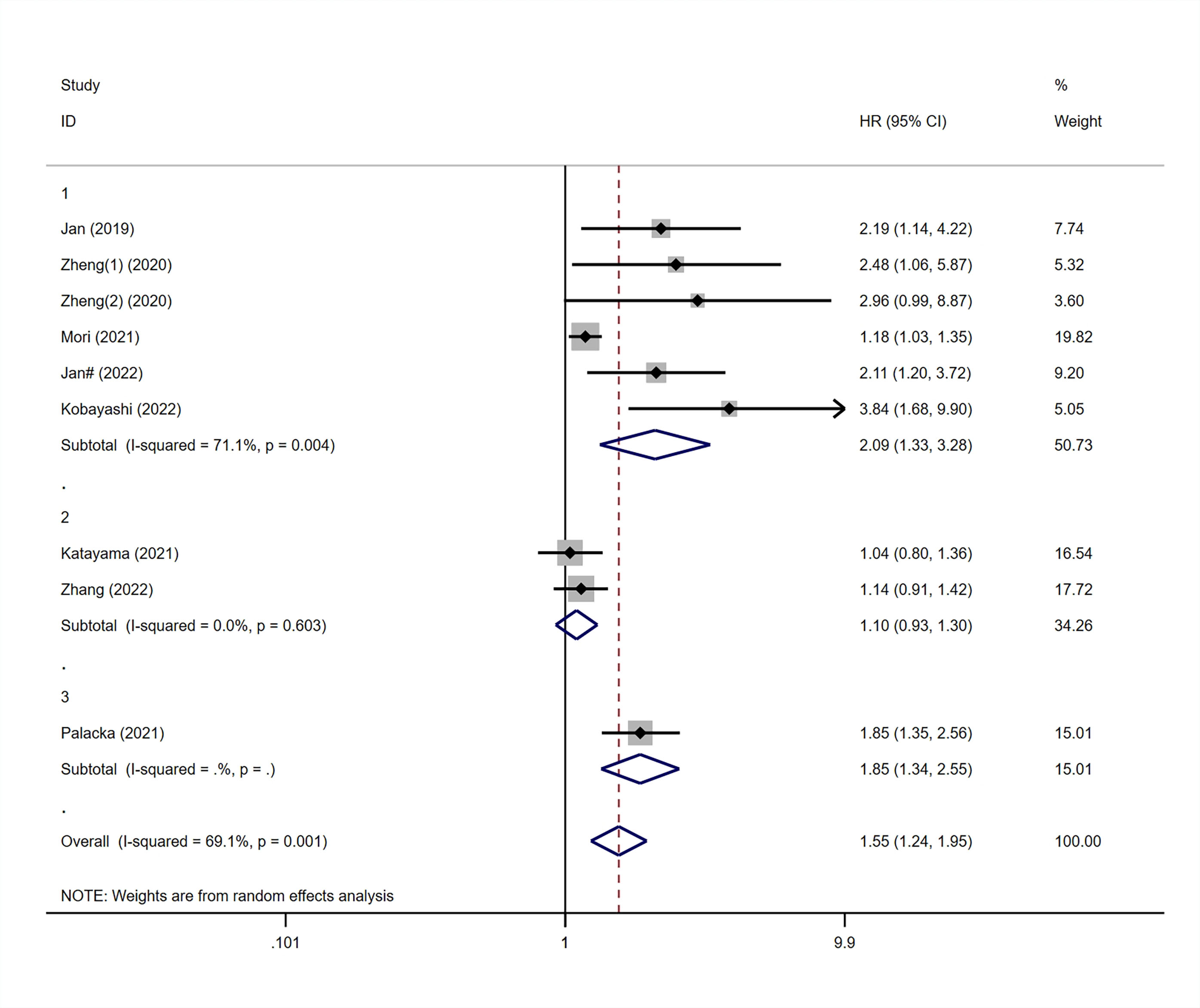** | **(D)**  **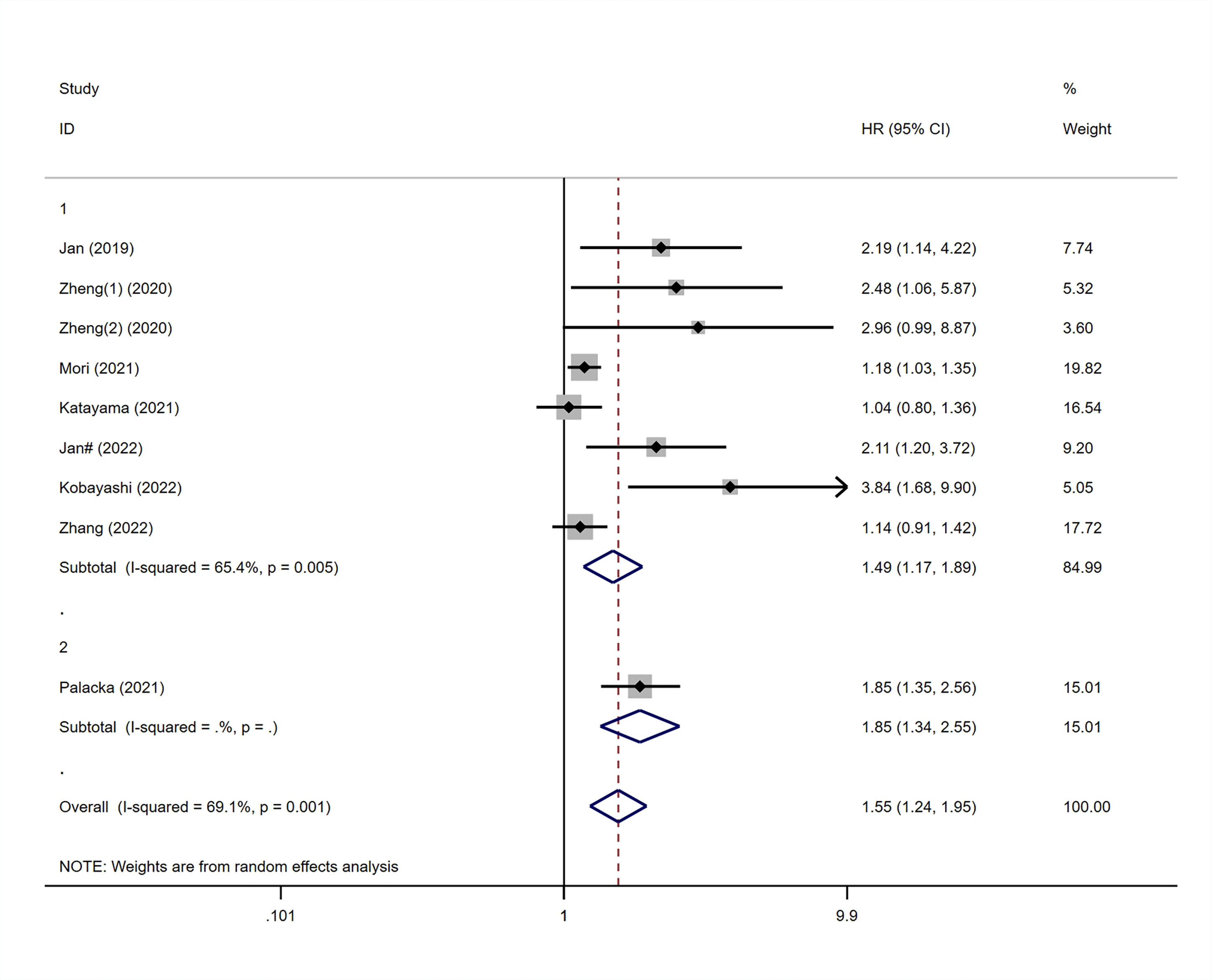** |
| **(E)**  **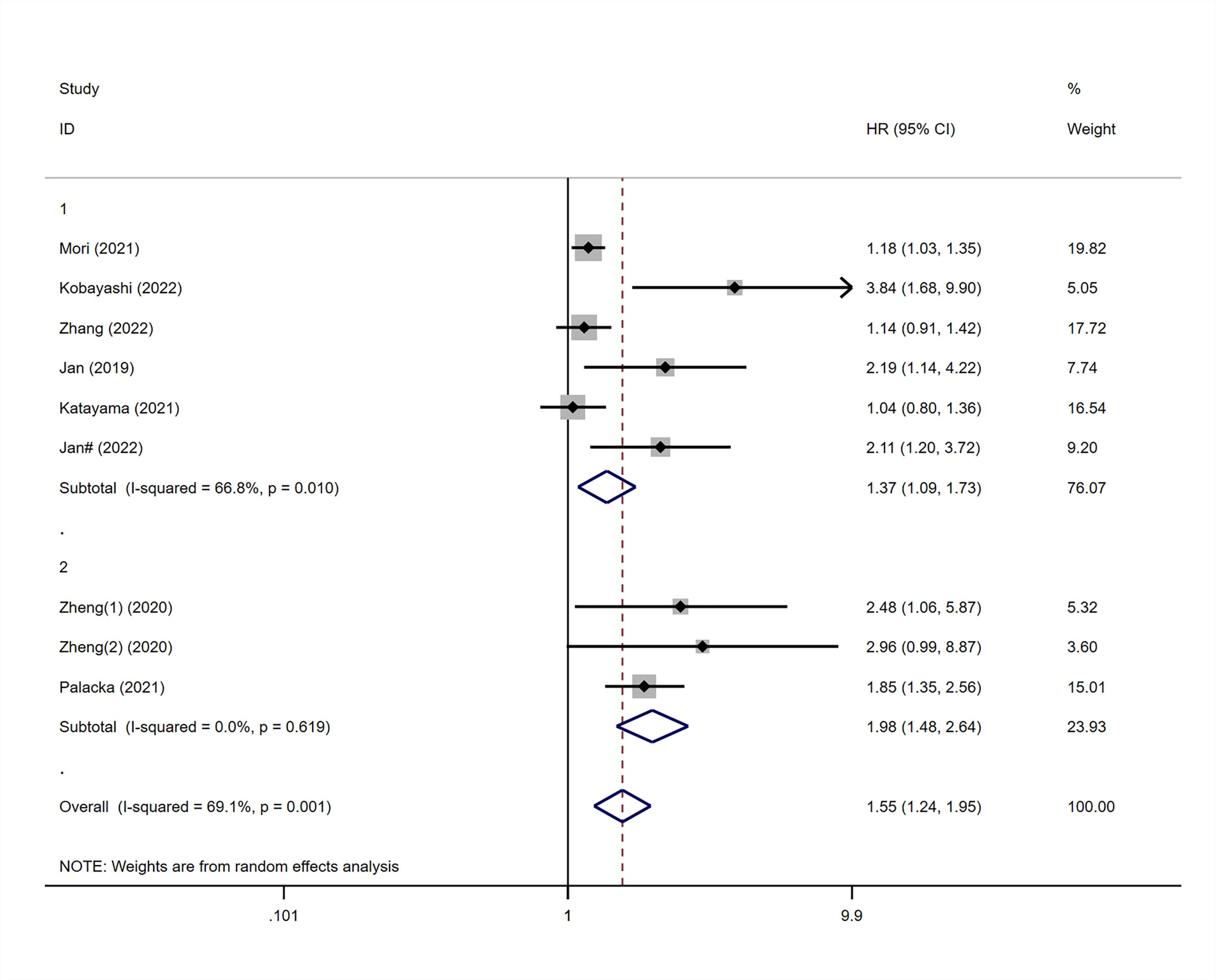** | **(F)**  **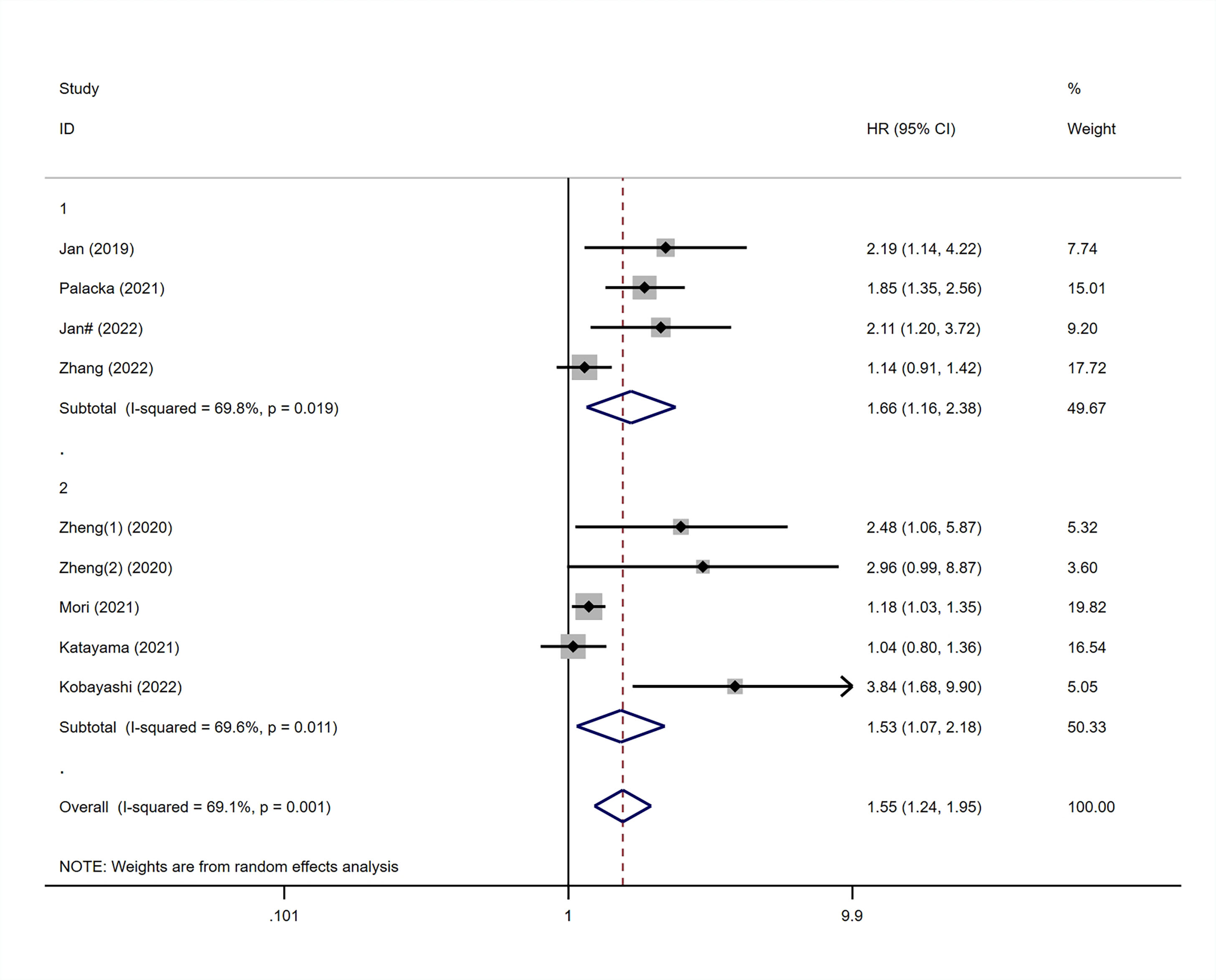** |

**Figure S1.** Subgroup analysis forest plot of the prognostic effect of SII on OS in UC. (A) region; (B) sample size; (C) cancer type; (D) treatment modality; (E) SII cut-off value; (F) NOS score.

| **(A)**  **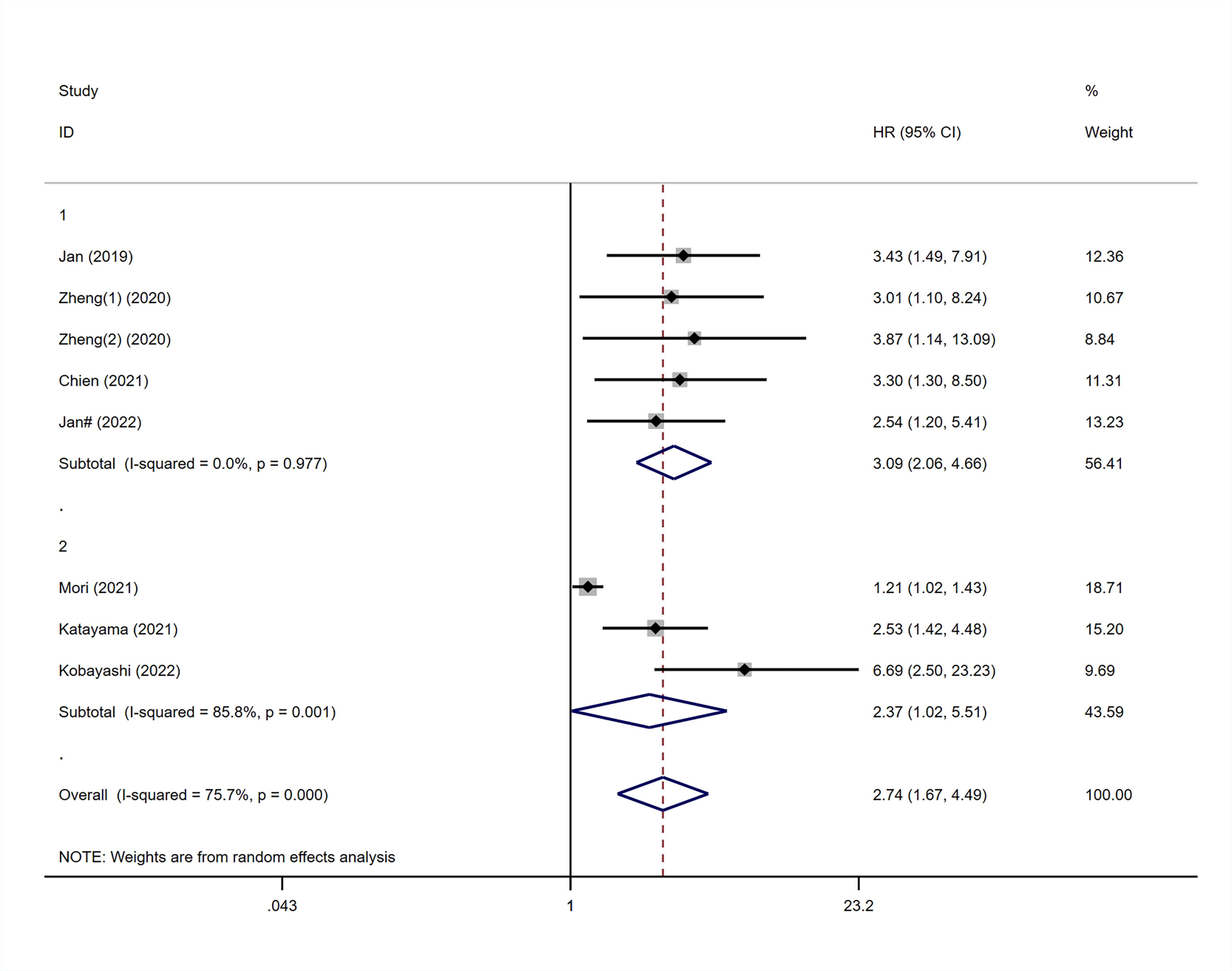** | **(B)**  **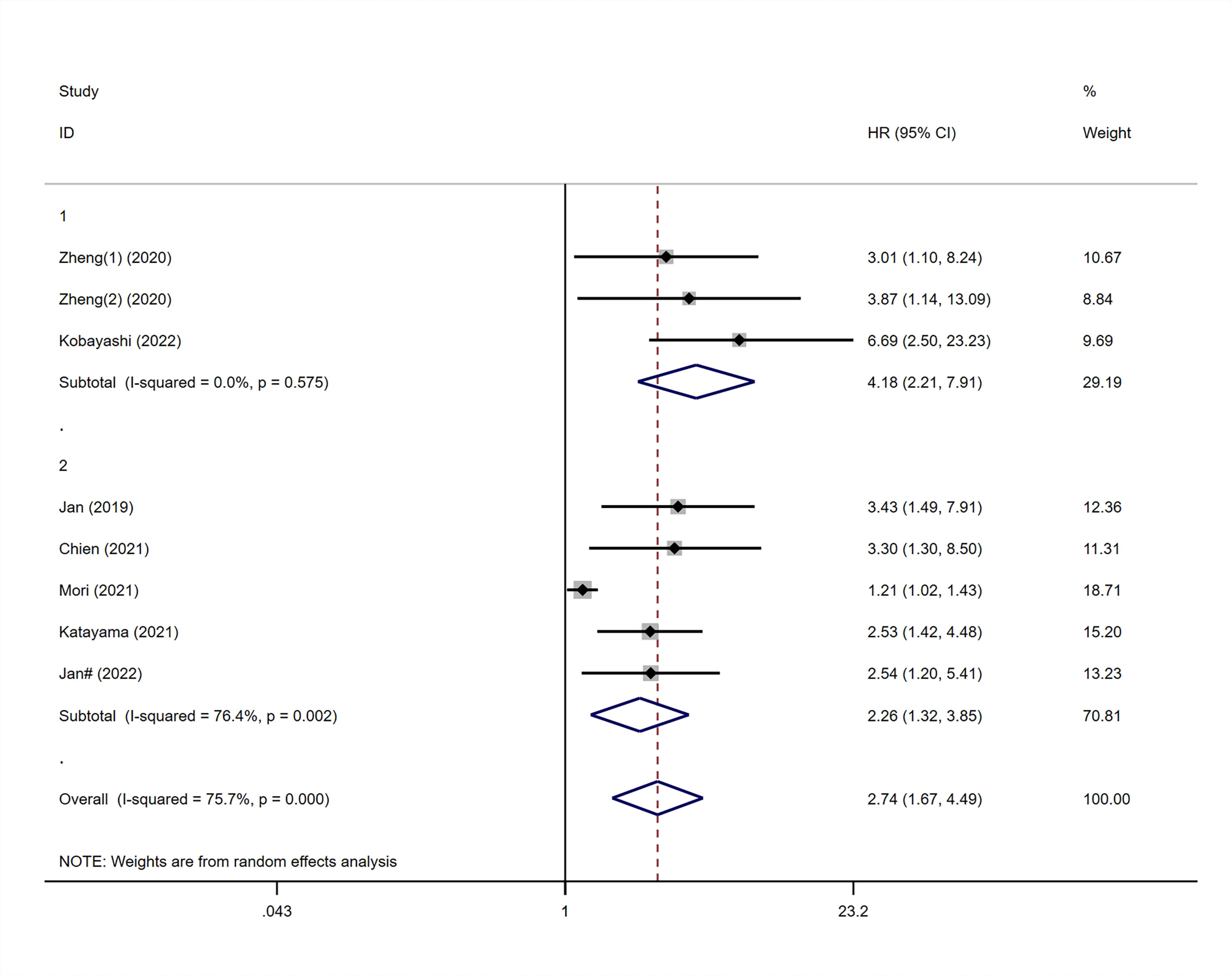** |
| --- | --- |
| **(C)**  **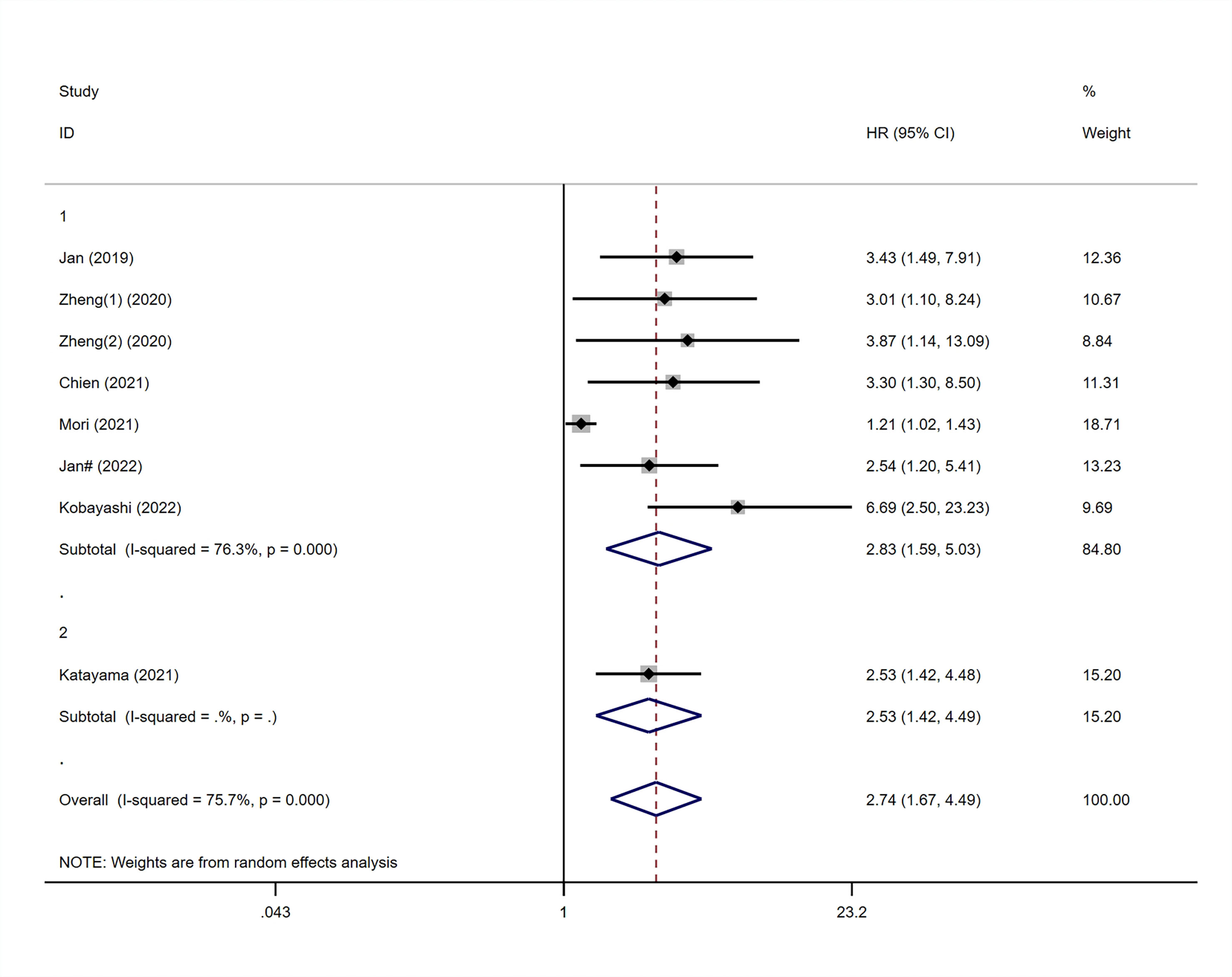** | **(D)**  **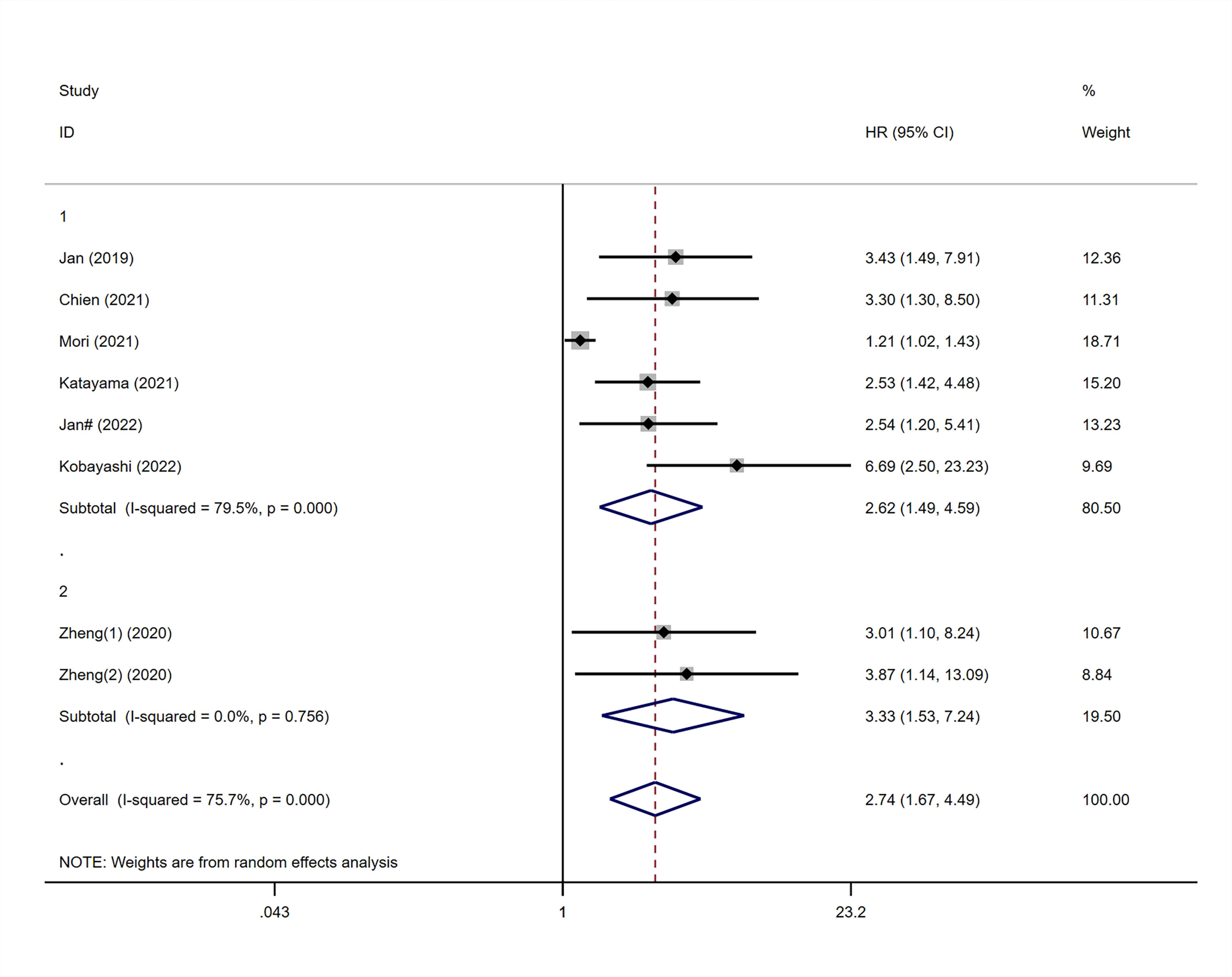** |
| **(E)**  **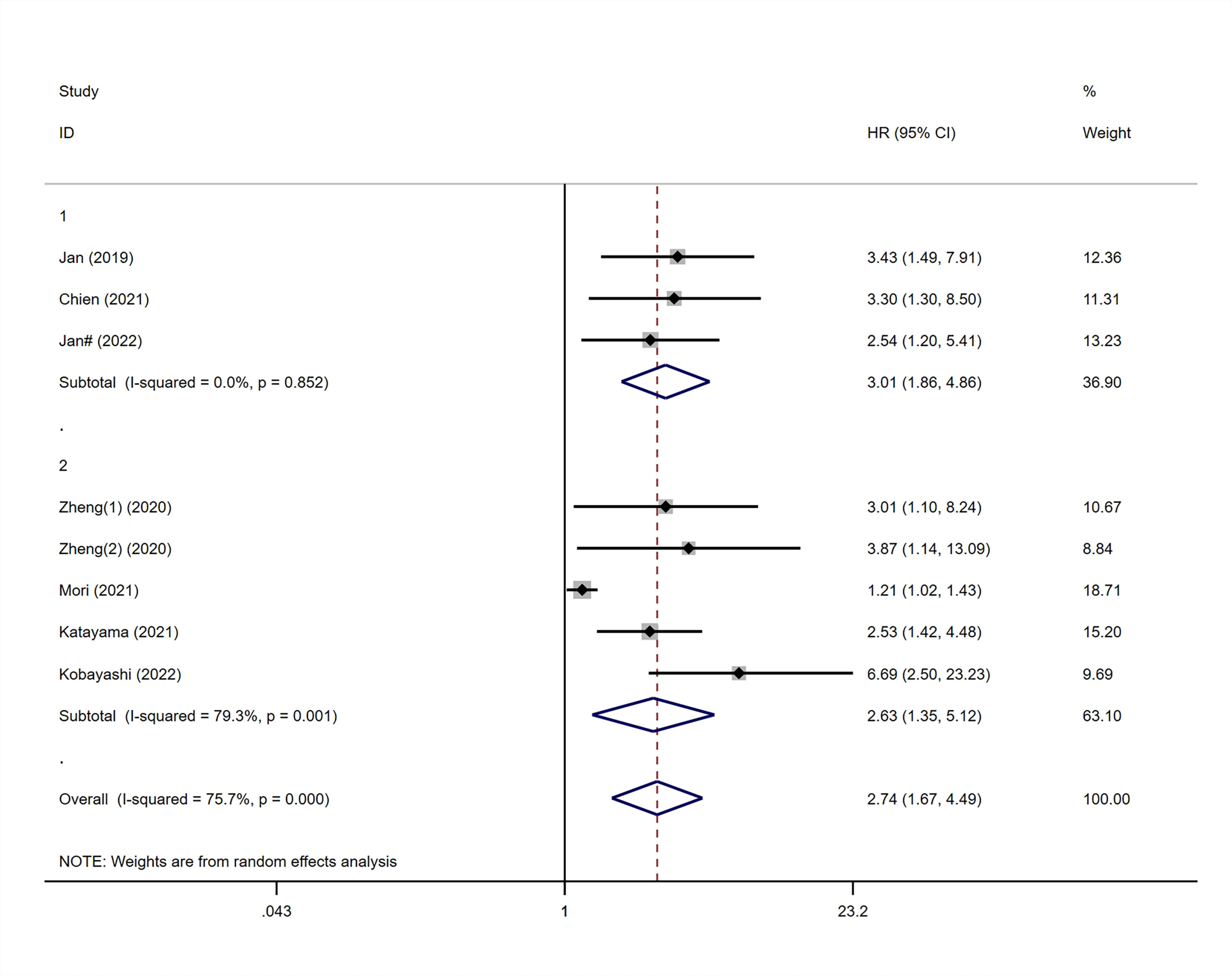** |  |

**Figure S2.** Subgroup analysis forest plot of the prognostic effect of SII on CSS in UC. (A) region; (B) sample size; (C) cancer type; (D) SII cut-off value; (E) NOS score.
